# Supplementary material for: Allosteric regulation and crystallographic fragment screening of SARS-CoV-2 NSP15 endoribonuclease
Source: Nucleic Acids Res. 2023 Apr 28;51(10):5255–70. doi: 10.1093/nar/gkad314 (PMC10250223; doi:10.1093/nar/gkad314)
Supplement: gkad314_Supplemental_Files [file gkad314_supplemental_files.zip › Supplementary datasets.zip!/81/Refine_9/01-BUSTER/Cycle-5/LIST.html]

Refining

**BUSTER 2.X**: A suite of programs for macromolecular structure refinement by maximum-likelihood and Bayesian methods  
Copyright © 1992-2020 Global Phasing Ltd.  
All rights reserved.  
Authors: G. Bricogne, E. Blanc, M. Brandl, C. Flensburg, P. Keller, W. Paciorek, P. Roversi, O. Smart, C. Vonrhein, T. Womack  

Please reference use of the software as:

- BUSTER-TNT 2.X, Global Phasing Ltd, Sheraton House, Cambridge CB3 0AX, UK

Version: 2.10.3 for linux64-ifort made on Sep 18 2020 at 01:21:52  
Licenced to: Diamond Light Source, Scientific Software  

```
---


### Parsing of the BUSTER cardfile

TITLE : autoBUSTER refinement

   Cell information

   Symmetry information

 Using CCP4 X-ray form factors.

   Missing structure chemical composition

 Using ATOM block for chemical composition.


   Shell information

### Parsing of the TNT cardfile


---


### Reading observations file

 Number of reflexions in input:  57108

      Number of accepted reflexions:  55835

      Number of rejected reflexions:      0

   Preparing complete.mtz containing:      57082 reflexions


   Reflexions in test set:            2859

   Reflexions in working set:        52976


---


### 1. Generating initial model

 2.10 * Shannon sampling rate. NX NY NZ:   288  288  240


  Effective Shannon rates are:    2.29    2.29    2.67


   Allocated BUFFER of size:     76.465 Mb.

   Initial Geometry Information


 No missing atoms declared


   Summary of model volumes

 No missing atoms declared: no prior computed


 Computing bulk solvent model scattering


 NCSMASK PDB radius for solvent mask:     2.15 Angstrom

 running PDBSET (setting cell and spacegroup P1) ... done
 running NCSMASK [1] (creating mask, SMOOTH 1.05084) ... done
 running MAPDUMP (to check if reduction to unit cell is needed) ... done
 running MAPMASK (converting mask to map) ... done

   Generation of babslv by theta-filter

   Electron density in frg mask:  0.420 Å-1

 FOBS  put on rough absolute scale by Wilson plot :
           K        =  0.50491E+02
           B        =    -16.171

 

### Cycle 0001: MaxLik scaling and refinement


   MaxLik Scaling Cycle 0001

   Wilson plot

   Statistics in resolution bins just after scaling
```

|  |  |
| --- | --- |
|  | - Rxpct factors plot- Rxpct factors table- <Log-Lik Gain>- Ln Sigma A- Correlation Coefficients |

```
   <Log-Likelihood> of null-hypothesis

Structure Factor Amplitudes


   Average Figure of Merit

 Switching off refinement of overall temperature factor


 Added B =    -0.5679 Å2 to all atoms in fragment


 Added B =    -0.5679 Å2 to B_SOLV

```
gelly refinement module
 Authors (2004-2009) O. S. Smart, C. Flensburg, W. Paciorek, C. Vonrhein, 
                     M. Brandl, T. O. Womack and G. Bricogne
         (2010-2011) O. S. Smart, C. Flensburg, W. Paciorek, C. Vonrhein, 
                     T. O. Womack and G. Bricogne
         (2012-2014) O. S. Smart, C. Flensburg, W. Paciorek, C. Vonrhein, 
                     and G. Bricogne
         (2015-2020) G. Bricogne, C. Flensburg, W. Paciorek, C. Vonrhein
 Copyright © 2004-2020 Global Phasing Ltd. 
 All rights reserved.
 gelly module compilation date Sep 18 2020 01:18:35
 
 gelly uses routines from TNT geometry. References: 
     Tronrud, D. E., Ten Eyck, L. F., Matthews, B. W. (1987)
     "An Efficient General-Purpose Least-Squares Refinement Program 
     for Macromolecular Structures". Acta Crystallogr A, 43:489-501.
     Tronrud, D. E. (1997). "The TNT Refinement Package", 
     in Macromolecular Crystallography, Part B, Eds Charlie Carter 
     and Robert Sweet, Methods in Enzymology, 277:306-319.
 wildcard matching function by Julian Robichaux http://www.nsftools.com

 No -random_seed flag found. Random number stream initialised using:
2147483562
2147483398

 Control flags set to:  -autoncs -hydrogens_input_occ2zero \
                     -hydrogens_output_occ2heavy -screen \
                     100 -screen_sigma \
                     3.0 -keeppdb \
                     1000000 -taomonitor_verbose \
                     2 
 

 ############### Starting setup via TNT routines

 Setting up tnt routines including license check:

                   TNT Stereochemistry Program
 Version: 5.22.3.0
 Licence for Diamond Light Source, Scientific Software is valid
 
 Using TNT geometry routines to load the tnt control file: gelly.tnt
 Using TNT routines to load initial coordinates from file fragment.cor
 Expecting 5526 atoms

 Found     2171 "NOTE BUSTER_RESIDUE_ATOM_DATA" cards
 Have copied information for    5526 atoms from TNT to gelly's store 
 
 The TNT WEIGHT cards used by this run:
 -----WEIGHT       BOND     2.0
 -----WEIGHT       ANGLE    2.0
 -----WEIGHT       IMPROPER 0.0
 -----WEIGHT       TORSION  2.0
 -----WEIGHT       PSEUDO   0.0
 -----WEIGHT       TRIGONAL 2.0
 -----WEIGHT       PLANE    5.0
 -----WEIGHT       CONTACT  5.0
 -----WEIGHT       BCORREL  10.0
 -----WEIGHT       CHIRAL   5.0
 -----WEIGHT       NCS      50.0
 -----WEIGHT       IDEAL    4.0
 -----WEIGHT       RFACTOR  8.52
 
 Dictionary versions picked up from NOTE BUSTER_RESTRAINT_DICT cards as: 
 ------ protgeo_eh99.dat (V1.6.4.17) 20191001  STANDARD AMINO ACID DICTIONARY. BONDS AND ANGLES FROM ENGH AND HUBER EH99. OTHER VALUES BASED ON PREVIOUS TNT OR TAKEN FROM CCP4. INCLUDES HYDROGEN ATOMS.
 ------ exoticaa.dat (V1.3.4.15) 20200327  COLLECTION OF NON-STANDARD AMINO ACIDS, MAINLY EH91 WITHOUT IDEAL DISTANCE INFO
 ------ nuclgeo.dat (V1.13.4.8) 20190909
 ------ bcorrel.dat (V1.15.10.2) 20190616
 ------ contact.dat (V1.15.12.10) 20191121
 ------ idealdist_contact.dat (V1.3.4.12) 20191001  IDEAL-DISTANCE CONTACT TERM DATA AS USED IN PROLSQ. VALUES USED HERE ARE BASED ON DATA HARVESTING OF PDB AS OF 20190109.
 ------ restraints for LIG (Unknown) from cif dictionary Z1741794237.cif
 ------ assume.dat (V1.9.4.2) 20180705
 
 Have picked up spacegroup name for pdb output as 'P 63      '
 Setting up Chain/Residue arrays
 
 Have command line specification -autoncs*
     Have found       1 NCS relations
     Pairwise matching residue number and type between chains: 
                    A        B    
          A         .      100%  
          B                  .   
       Table key--                                                       
          all   : two chains have 100% match and same number of residues 
         multi  : ditto and they contribute to a multi chain ncs cluster 
          ***%  : two chains have >80% matching residue number/type      
           -    : chains appear to be unrelated                          
      chain A     have identified ncs to     1 other chains. So will weight each autoncs by (       6.666/#)=      6.6660
      chain B     have identified ncs to     1 other chains. So will weight each autoncs by (       6.666/#)=      6.6660
     For .Gelly cards produced see below - after pruning
 
 Setting up BUSTER_SET groups
 Have found    89 BUSTER_SET cards
 Now silently applying automatic/dictionary set definitions. For a verbose listing use '-verbose_set' flag.
 Applying user defined set definitions:
 =====NOTE BUSTER_SET ncsautoXcld = Water --> set 'ncsautoXcld' now has     0 atoms 
 =====NOTE BUSTER_SET ncsautoABset = Chain_A \ ncsautoXcld --> set 'ncsautoABset' now has  2768 atoms 
 Have defined    47 sets: 
      set:                 'All' has   5526 atoms in    699 residues - automatically defined
      set:               'Empty' has      0 atoms in      0 residues - automatically defined
      set:            'ExoticAA' has      0 atoms in      0 residues - defined by dictionary file
      set:           'ExoticNUC' has      0 atoms in      0 residues - defined by dictionary file
      set:              'NotHET' has   5516 atoms in    698 residues - defined by dictionary file
      set:             'Chain_A' has   2768 atoms in    350 residues - automatically defined
      set:             'Chain_B' has   2758 atoms in    349 residues - automatically defined
      set:               'Water' has      0 atoms in      0 residues - defined by dictionary file
      set:          'StdProtein' has   5516 atoms in    698 residues - defined by dictionary file
      set:             'Protein' has   5516 atoms in    698 residues - defined by dictionary file
      set:                'Back' has   2794 atoms in    698 residues - defined by dictionary file
      set:                'Side' has   2722 atoms in    652 residues - defined by dictionary file
      set:        'RnaCanonical' has      0 atoms in      0 residues - defined by dictionary file
      set:        'DnaCanonical' has      0 atoms in      0 residues - defined by dictionary file
      set:     'RnaDnaCanonical' has      0 atoms in      0 residues - defined by dictionary file
      set:     'DnaRnaCanonical' has      0 atoms in      0 residues - defined by dictionary file
      set:         'RnaExtended' has      0 atoms in      0 residues - defined by dictionary file
      set:         'DnaExtended' has      0 atoms in      0 residues - defined by dictionary file
      set:      'RnaDnaExtended' has      0 atoms in      0 residues - defined by dictionary file
      set:      'DnaRnaExtended' has      0 atoms in      0 residues - defined by dictionary file
      set:                 'Dna' has      0 atoms in      0 residues - defined by dictionary file
      set:                 'Rna' has      0 atoms in      0 residues - defined by dictionary file
      set:              'RnaDna' has      0 atoms in      0 residues - defined by dictionary file
      set:              'DnaRna' has      0 atoms in      0 residues - defined by dictionary file
      set:             'Nucleic' has      0 atoms in      0 residues - defined by dictionary file
      set:             'Polymer' has   5516 atoms in    698 residues - defined by dictionary file
      set: 'BufferWeichenberger' has      0 atoms in      0 residues - defined by dictionary file
      set:    'BufferAdditional' has      0 atoms in      0 residues - defined by dictionary file
      set:              'Buffer' has      0 atoms in      0 residues - defined by dictionary file
      set:         'MetalAlkali' has      0 atoms in      0 residues - defined by dictionary file
      set:  'MetalAlkalineEarth' has      0 atoms in      0 residues - defined by dictionary file
      set:     'MetalTransition' has      0 atoms in      0 residues - defined by dictionary file
      set: 'MetalPostTransition' has      0 atoms in      0 residues - defined by dictionary file
      set:    'MetalLanthanides' has      0 atoms in      0 residues - defined by dictionary file
      set:               'Metal' has      0 atoms in      0 residues - defined by dictionary file
      set:         'SugarAlphaD' has      0 atoms in      0 residues - defined by dictionary file
      set:         'SugarAlphaL' has      0 atoms in      0 residues - defined by dictionary file
      set:               'Sugar' has      0 atoms in      0 residues - defined by dictionary file
      set:               'Other' has     10 atoms in      1 residues - defined by dictionary file
      set:            'Hydrogen' has      0 atoms in      0 residues - defined by dictionary file
      set:         'nonHydrogen' has   5526 atoms in    699 residues - defined by dictionary file
      set:             'OccZero' has      0 atoms in      0 residues - defined by dictionary file
      set:          'OccNonZero' has   5526 atoms in    699 residues - defined by dictionary file
      set:             'OccFull' has   5484 atoms in    696 residues - defined by dictionary file
      set:          'OccNonFull' has     42 atoms in      3 residues - defined by dictionary file
      set:         'ncsautoXcld' has      0 atoms in      0 residues - user specified
      set:        'ncsautoABset' has   2768 atoms in    350 residues - user specified
 No command line arguments '-occ_allhet' or '-occ_restyp' so will not setup automated occcupancy refinement
 
 Interpreting CONSTANT and FREE cards: 
 =====CONSTANT     OCC
 Have found and interpreted      1 CONSTANT cards in the TNT input. 
 Before any COMBINE - Number of atoms with fixed XYZ =     0
 Before any COMBINE - Number of atoms with fixed B   =     0
 Before any COMBINE - Number of atoms with fixed OCC =  5526
 Before any COMBINE - Number of atoms with fixed BIJ =     0
                            cf total number of atoms =  5526
 
 There are no COMBINE cards to be found in the TNT card input 
 
 There are no NOTE BUSTER_COMBINE_RES cards to be found in the TNT card input 

 ############### Working out full connectivity list


 ############### Working out equivalent atoms


 Residue with most atoms given below:
        A|58:N(TRP)         
        A|58:CA(TRP)        
        A|58:CB(TRP)        
        A|58:CG(TRP)        
        A|58:CD1(TRP)       
        A|58:NE1(TRP)       
        A|58:CE2(TRP)       
        A|58:CD2(TRP)       
        A|58:CE3(TRP)       
        A|58:CZ3(TRP)       
        A|58:CH2(TRP)       
        A|58:CZ2(TRP)       
        A|58:C(TRP)         
        A|58:O(TRP)         


 Have no '-equiv_check_plus' argument - so will take atom type into
 account when checking for equivalence of otherwise identical atoms.
 

 ############### Looking at dihedrals


 ############### Looking at chirals

 NOTE BUSTER_CHIRAL_ANGLECUT card found - read value=    -31.00 degs from it
 NOTE BUSTER_CHIRAL_SIGMA card found - read value=      1.70 degs from it
 RANGE allowed for B variables in refinement:   from      3.00000 to    300.00000

 ############### Preparing for PDB output


 ############### Processing extra restraints explicitly defined

 No 'NOTE BUSTER_TARGET' cards or -target?? args found (used to read coordinates files for soft ncs to external)
 No 'NOTE BUSTER_OCCSUM'     cards found - so not using sum of occupancy restraints
 No 'NOTE BUSTER_DISTANCE'   cards found - so not setting up any additional atom-atom distance restraints
 No 'NOTE BUSTER_UTILANGLE'  cards found - so not setting up any additional restraints on any angle
 No 'NOTE BUSTER_UTILTOR'    cards found - so not setting up any additional sinusoidal restraints on torsions
 
 --- Have some similarity definitions 
     Similarity restraints on RMSD use superposition routines from: 
     Coutsias, E.A., Seok, C., Dill, K.A.(2004)
     "Using quaternions to calculate RMSD", J. Comput. Chem., 25:1849-1857 

 Have found    1 'NOTE BUSTER_SIM_DEFINE' cards 
     Maximum number of chains involved in any SIM group (excluding the template)=     1


 ########## Interpreting card: NOTE BUSTER_SIM_DEFINE ncsautoAB ncsautoABset B
     Number of atoms in template set=  2768
     Number of related chains (other than template) in SIM set=    1
     List of chains to be restrained (excluding template)=   B   
     Pairwise RMSD, XYZ in Angstroms, for SIM group: ncsautoAB
                 A         B   
     A         -----     ----- 
     B         0.652     ----- 
     Overall RMSD,XYZ for the SIM group is     0.652 Angstroms
     Pairwise rms deviation of [individual B's minus their set/chain average] in Angs**2
                 A         B         Bavg
     A         -----     -----     90.30
     B         35.13     -----     64.79
     Overall RMSD,B   for the SIM group is    35.131 Angs**2
     The number of common atoms for the above fits:
                 A         B   
     A         -----     ----- 
     B          2758     ----- 
     (compared to the template set that has    2768 atoms in it)
     List of atoms that are absent in one or more chains for this group: 
                                    A    B   
                    C01     401     +    -
                    C02     401     +    -
                    C03     401     +    -
                    N04     401     +    -
                    C05     401     +    -
                    C06     401     +    -
                    N07     401     +    -
                    C08     401     +    -
                    C09     401     +    -
                    O10     401     +    -
     (key  +  atom present for this chain 
           -  atom absent  for this chain)
 transforms for each of the superpositions orthogonal matrix r11 r12 r13 r21 r22 r23 r31 r32 r33 tx ty tz (PDBSET syntax): 
TRANS_AB -0.873353 -0.486360  0.026627 -0.486316  0.873741  0.008537 -0.027417 -0.005493 -0.999609    44.151   -12.859    15.147
 reverse transforms 
TRANS_BA -0.873353 -0.486316 -0.027417 -0.486360  0.873741 -0.005493  0.026627  0.008537 -0.999609    32.721    32.792    14.075
 
 Setting up LSSR interatomic distance restraints
 Interpreting card: NOTE BUSTER_SIM_RESTRAIN_LSSR ncsautoAB 6.666
        setting up interatomic distance pair restraints with:
        Dmin=         0.00
        Dmax=         5.50
        sigma=        0.20
        Vmax=         3.00
        weight=       6.67
        number of distance pairs defined for this set is      34346
 Have not supplied either '-sim_swap_equiv' or '-sim_swap_equiv_plus' argument 
   so will not try swaping equivalent atoms to improve NCS and/or target agreement.
 
 NCS by LSSR outlier analysis
 -autoncs option automatic NCS prune routine -------- 
 Residues pruned out by gradient condition:
           A|23      A|27      A|32      A|33      A|34      A|37      A|39
          A|147     A|148     A|149     A|150     A|151     A|157     A|158
          A|159     A|160     A|168     A|169     A|172     A|173     A|174
          A|175     A|185     A|186     A|187     A|188     A|216     A|220
          A|229     A|257     A|259     A|260     A|261     A|264     A|306
          A|307     A|308     A|309     A|320     A|322     A|325     A|326
          A|327     A|328     A|346      B|23      B|27      B|32      B|33
           B|34      B|37      B|39     B|147     B|148     B|149     B|150
          B|151     B|157     B|158     B|159     B|160     B|168     B|169
          B|172     B|173     B|174     B|175     B|185     B|186     B|187
          B|188     B|216     B|220     B|229     B|257     B|259     B|260
          B|261     B|264     B|306     B|307     B|308     B|309     B|320
          B|322     B|325     B|326     B|327     B|328     B|346
  
 Residues pruned out by function/max condition:
          A|149!    A|169!    A|170     A|171     A|172!    A|173!    A|187!
          A|188!    A|220!    A|259!    A|346!   A|ENDA     B|149!    B|169!
          B|170     B|171     B|172!    B|173!    B|187!    B|188!    B|220!
          B|259!    B|346!   B|ENDA 
  
 Total number of residues for which LSSR restraints have been pruned out is       96 out of      698 participating
 Number of LSSR restraints pruned out is      6132 this is   17.9% of the total           34346
  
 -autoncs .Gelly cards suitable for adaptation for manual definition/pruning
           (note auto pruning is done in a slightly different way so exact results may differ slightly)
 -autoncs option .Gelly cards also written to file  auto_gelly_cards.txt
NOTE BUSTER_SET ncsautoXcld = Water    # card added - produced by -autoncs
NOTE BUSTER_SET ncsautoXcld = ncsautoXcld + { A|23 A|27 A|32 A|33 A|34 A|37 }    # card added - produced by -autoncs
NOTE BUSTER_SET ncsautoXcld = ncsautoXcld + { A|39 A|147 A|148 A|149 A|150 }    # card added - produced by -autoncs
NOTE BUSTER_SET ncsautoXcld = ncsautoXcld + { A|151 A|157 A|158 A|159 A|160 }    # card added - produced by -autoncs
NOTE BUSTER_SET ncsautoXcld = ncsautoXcld + { A|168 A|169 A|170 A|171 A|172 }    # card added - produced by -autoncs
NOTE BUSTER_SET ncsautoXcld = ncsautoXcld + { A|173 A|174 A|175 A|185 A|186 }    # card added - produced by -autoncs
NOTE BUSTER_SET ncsautoXcld = ncsautoXcld + { A|187 A|188 A|216 A|220 A|229 }    # card added - produced by -autoncs
NOTE BUSTER_SET ncsautoXcld = ncsautoXcld + { A|257 A|259 A|260 A|261 A|264 }    # card added - produced by -autoncs
NOTE BUSTER_SET ncsautoXcld = ncsautoXcld + { A|306 A|307 A|308 A|309 A|320 }    # card added - produced by -autoncs
NOTE BUSTER_SET ncsautoXcld = ncsautoXcld + { A|322 A|325 A|326 A|327 A|328 }    # card added - produced by -autoncs
NOTE BUSTER_SET ncsautoXcld = ncsautoXcld + { A|346 A|ENDA }    # card added - produced by -autoncs
NOTE BUSTER_SET ncsautoABset = Chain_A \ ncsautoXcld    # card added - produced by -autoncs
NOTE BUSTER_SIM_DEFINE ncsautoAB ncsautoABset B    # card added - produced by -autoncs
NOTE BUSTER_SIM_RESTRAIN_LSSR ncsautoAB 6.666    # card added - produced by -autoncs

 LSSR function contribution before pruning     40173.1
                             after pruning      7122.1
 Graph of which residues pruned from LSSR: ncs_pruned.mtv
 Graph of LSSR function outliers ncs_function_outliers.mtv
 Graph of LSSR gradient outliers ncs_gradient_outliers.mtv
 LSSR - analysis of distance difference distribution for NCS
         DeltaD less than 0.5 Angs:  proportion of population= 99.14% average DeltaD of subset     0.012 Angs
 Histogram of LSSR deltaD distribution ncs_histogram.mtv
 
 Interpreting card: NOTE BUSTER_TLS_SET tls1 { A|* }
 Interpreting card: NOTE BUSTER_TLS_SET tls2 { B|* }
 Interpreting card: NOTE BUSTER_TLS_SET tls1 { A|* }
 Interpreting card: NOTE BUSTER_TLS_T   tls1     -1.64775E-01 -3.36840E-02  2.15737E-01 -3.93502E-02 -3.35143E-02 -2.21740E-02
 Interpreting card: NOTE BUSTER_TLS_L   tls1      1.33783E+00  1.61817E+00  1.44183E+00 -3.13773E-01  8.90801E-01 -8.51731E-01
 Interpreting card: NOTE BUSTER_TLS_S   tls1      1.51047E-01 -2.84462E-01  3.13261E-01  6.85951E-02 -1.30648E-01 -2.56570E-01  7.59762E-02  1.23108E-01
 Interpreting card: NOTE BUSTER_TLS_O   tls1      2.31588E+01 -6.71772E+01  1.06797E+01
 Interpreting card: NOTE BUSTER_TLS_FIX tls1 ALL
 Interpreting card: NOTE BUSTER_TLS_SET tls2 { B|* }
 Interpreting card: NOTE BUSTER_TLS_T   tls2     -2.34737E-01 -9.54492E-02  3.04843E-01  1.30665E-02 -3.15132E-02  3.78123E-02
 Interpreting card: NOTE BUSTER_TLS_L   tls2      1.51690E+00  1.05873E+00  1.03399E+00  6.62845E-01  9.72762E-01  7.91924E-01
 Interpreting card: NOTE BUSTER_TLS_S   tls2      8.67167E-04  4.13351E-02 -2.05843E-01 -1.01273E-01 -1.11597E-01  4.88749E-02  1.27152E-01 -6.61129E-02
 Interpreting card: NOTE BUSTER_TLS_O   tls2     -2.22253E+01 -7.89989E+01  3.21640E+00
 Interpreting card: NOTE BUSTER_TLS_FIX tls2 ALL


 TLS information. Total number of groups:     2


 Group   Natoms    FixRB   FixTLS   Group Tag    Group Spec
     1     2768      Y        Y     tls1    { A|* }
     2     2758      Y        Y     tls2    { B|* }
 
NOTE BUSTER_TLS_SET tls1 { A|* }
NOTE BUSTER_TLS_T   tls1     -1.64775E-01 -3.36840E-02  2.15737E-01 -3.93502E-02 -3.35143E-02 -2.21740E-02
NOTE BUSTER_TLS_L   tls1      1.33783E+00  1.61817E+00  1.44183E+00 -3.13773E-01  8.90801E-01 -8.51731E-01
NOTE BUSTER_TLS_S   tls1      1.51047E-01 -2.84462E-01  3.13261E-01  6.85951E-02 -1.30648E-01 -2.56570E-01  7.59762E-02  1.23108E-01
NOTE BUSTER_TLS_O   tls1      2.31588E+01 -6.71772E+01  1.06797E+01
NOTE BUSTER_TLS_FIX tls1 ALL
 
NOTE BUSTER_TLS_SET tls2 { B|* }
NOTE BUSTER_TLS_T   tls2     -2.34737E-01 -9.54492E-02  3.04843E-01  1.30665E-02 -3.15132E-02  3.78123E-02
NOTE BUSTER_TLS_L   tls2      1.51690E+00  1.05873E+00  1.03399E+00  6.62845E-01  9.72762E-01  7.91924E-01
NOTE BUSTER_TLS_S   tls2      8.67167E-04  4.13351E-02 -2.05843E-01 -1.01273E-01 -1.11597E-01  4.88749E-02  1.27152E-01 -6.61129E-02
NOTE BUSTER_TLS_O   tls2     -2.22253E+01 -7.89989E+01  3.21640E+00
NOTE BUSTER_TLS_FIX tls2 ALL


 TLS: mmCIF group specification begin


#
loop_
_pdbx_refine_tls_group.id
_pdbx_refine_tls_group.refine_tls_id
_pdbx_refine_tls_group.beg_PDB_ins_code
_pdbx_refine_tls_group.beg_auth_asym_id
_pdbx_refine_tls_group.beg_auth_seq_id
_pdbx_refine_tls_group.end_PDB_ins_code
_pdbx_refine_tls_group.end_auth_asym_id
_pdbx_refine_tls_group.end_auth_seq_id
_pdbx_refine_tls_group.selection
_pdbx_refine_tls_group.selection_details
   1   1 . A   -1 . A  346 . '{ A|* }'
   2   1 . A  401 . A  401 . '{ A|* }'
   3   2 . B   -1 . B  346 . '{ B|* }'
#


 TLS: mmCIF group specification end


 Interpreting card: NOTE BUSTER_ANISO_SET Empty
 Interpreting card: NOTE BUSTER_ANISO_WEIGHT SCA  16.0
 Interpreting card: NOTE BUSTER_ANISO_WEIGHT SPH   0.6    0.6
 Interpreting card: NOTE BUSTER_ANISO_WEIGHT SIM   7.8    4.8    2.6    0
 Interpreting card: NOTE BUSTER_ANISO_WEIGHT RIG  21.5   21.5    3.7    0


 After TLS and ANISO: Number of atoms with fixed/combined xyz =     0
 After TLS and ANISO: Number of atoms with fixed/combined B   =     0
 After TLS and ANISO: Number of atoms with fixed/combined OCC =  5526
 After TLS and ANISO: Number of atoms with fixed/combined BIJ =  5526
 After TLS and ANISO: Number of atoms in a TLS group          =  5526
                                     cf total number of atoms =  5526
 

 ############### Setup state vector

 No free_vect_ulim_xyz argument found: using default of  100.000
 No free_vect_llim_xyz argument found: using default of -100.000
 Number of parameters added for TLS description                 =       0
 Number of parameters to describe molecular conformation        =   22104

 ############### Setup free vector

 Number of free parameters to be adjusted in refinement   =   22104

 ############### Setting up own geometry


 No command line argument '-tnt_fn' so will use gelly to do geometry function calculations

 ============= Call TNT functions


 ============= Get TNT symmetry information

 
      Getting symmetry operators from TNT. 
          gelly will classify symmetry using pdb-like convention: 
               SYMOP   SYMMETRY
              NNNMMM   OPERATOR
                1555   X,Y,Z
                2555   -Y,X-Y,Z
                3555   -X+Y,-X,Z
                4555   -X,-Y,1/2+Z
                5555   Y,-X+Y,1/2+Z
                6555   X-Y,X,1/2+Z
          where NNN -> operator number and MMM -> translation vector
 

 ============= Own Gelly geometry/restraints setup


 ----------- Identify bond-types


    Setting up GEOMBONDREC_TYPE_HASH* to be able to map a given bond type (e.g.
    "single") to an internal integer (GBOND_TYPE=1) etc.

    Using default  GEOMBONDREC_TYPE_HASH_STR='SING* 1 DOUB* 2 TRIP* 3 AROM* 4 DELO* 4 METAL* 5':
    to override default use a NOTE BUSTER_GEOMBONDREC_TYPE_HASH_STR gelly card.

 ----------- Classify torsion into peptide omega and other


 ----------- Setup of non-bonded terms

 Ideal-distance contact term setup:
    Restraint function constants (normally these values are from NOTE BUSTER_IDEAL_CONTACT_* cards)
    Sigma for general contacts      0.200 Angs
    Sigma for 1-4 contacts          0.100 Angs (contacts across a torsion)
    Sigma for 1-5 contacts          0.150 Angs
    Sigma for 1-N contacts          0.200 Angs
    Sigma for HBOND pairs           0.100 Angs (contacts between HB_types A and D or B's)
    Contact distance increment for each atom in HBOND          -0.150 Angs 
    Contact distance increment for H-bond acceptor              0.100 Angs 
    Contact distance increment for 1-4 with an O atom          -0.050 Angs 
    Contact distance increment for 1-4 with an N atom          -0.180 Angs 
    Contact distance increment for 1-4 with an C atom          -0.370 Angs 
    Contact distance increment for 1-4 with an S atom          -0.150 Angs 
    Contact distance increment for 1-4 with an P atom          -0.150 Angs 
    Contact distance increment for 1-4 with any other atom     -0.150 Angs 
    All atoms matched for CCP4 atom type.
    Number of CCP4 atom types used is         22 (H-bound and not H-bound counted separately)

 Setting up short/ideal-distance contacts
   Using gelly special position handling. (For TNT instead rerun with argument '-special_tnt')

   No command line argument '-special_all' specified. We will auto-generate
   EXCLUDE cards only for atom-atom contacts across symmetry (i.e. on special
   position) when this involves a contact within a residue to itself.

   Have set up non-bonded exclusions - 1-3 contacts
   Atom A|27:CA         makes the most 1-3 contacts:  6

   Have set up non-bonded exclusions - 1-4 contacts
   Atom A|27:N          makes the most 1-4 contacts:  7

   Have set up non-bonded exclusions - 1-5 contacts
   Atom A|28:C.A        makes the most 1-5 contacts: 10

 ############################################################################################################
 ###################################################### setting up contact terms
 ############################################################################################################


 ============= Call Gelly versions of TNT functions

 WARNING: 1-4 contact without 1-(2,3)-4 data for A|401:C01(LIG)       and A|401:C08(LIG)       X-X
 WARNING: 1-4 contact without 1-(2,3)-4 data for A|401:C02(LIG)       and A|401:C05(LIG)       X-X
 WARNING: 1-4 contact without 1-(2,3)-4 data for A|401:C03(LIG)       and A|401:C06(LIG)       X-X
 WARNING: 1-4 contact without 1-(2,3)-4 data for A|401:N04(LIG)       and A|401:N07(LIG)       X-X
 WARNING: 1-4 contact without 1-(2,3)-4 data for A|401:C06(LIG)       and A|401:C09(LIG)       X-X

       TERM      weight      number      TNT_fn    Gelly_fn        diff
       BOND       2.000        5642    1405.847    1405.847       0.000
       ANGL       2.000        7656    4980.579    4980.579       0.000
       IMPR       0.000           0       0.000       0.000       0.000
       TORS       2.000        1964    2887.613    2887.613      -0.000
       TRIG       2.000           0       0.000       0.000       0.000
       PLAN       5.000         958    2388.862    2388.862       0.000
       CONT       5.000           0       8.042       0.000       8.042
       BCOR      10.000        5632    7476.489    6012.894    1463.595


 ============= Call Gelly geometry functions

   Switching torsion functional form to sinusoidal rather than split harmonic functional.
      if you want this not to happen then rerun with '-torsharm'
      Weight for sinusoidal torsion=     2.000
   BCOR function/gradient will be split between H and non-H atoms (as for any atom pair). Use '-bcorrel_all_in_hydrogens' to change this.

 ----------- Fix B-correlation restraints

 
 Checking for bonds that do not have a bcorrel restraint defined
 We will wipe all existing BCORrels if OCC=0.0 atom(s) is/are involved. This can be' switched off via the '-bcorrel_nowipe_zeroocc' flag.
 We will wipe all existing BCORrels if only non-hydrogen atom(s) with fixed B(iso) are involved. This can be switched off via the '-bcorrel_nowipe_fixb_nonhydrogens' flag.
 We will keep all existing BCORrels if hydrogen atom(s) with fixed B(iso) is/are involved. This can be switched off via the '-bcorrel_wipe_fixb_hydrogens' flag.
 No -bcorrel_missing_sigma found. So will set sigma to     2.5 Ang**2 for any bond with no existing BCORrel
 No -bcorrel_missing_sigma_hydrogens found. So will set sigma to     1.0 Ang**2 for any bond with no existing BCORrel involving hydrogens

 No active BCORels found involving OCC=0.0 atom(s)


 No active BCORels found involving fixed-B (only) non-hydrogen atom(s)

    add a default BCOR for bond A|401:C01(LIG) A|401:C02(LIG)
    add a default BCOR for bond A|401:C02(LIG) A|401:C03(LIG)
    add a default BCOR for bond A|401:C02(LIG) A|401:N07(LIG)
    add a default BCOR for bond A|401:C03(LIG) A|401:N04(LIG)
    add a default BCOR for bond A|401:N04(LIG) A|401:C05(LIG)
    add a default BCOR for bond A|401:C05(LIG) A|401:C06(LIG)
    add a default BCOR for bond A|401:C06(LIG) A|401:N07(LIG)
    add a default BCOR for bond A|401:N07(LIG) A|401:C08(LIG)
    add a default BCOR for bond A|401:C08(LIG) A|401:C09(LIG)
    add a default BCOR for bond A|401:C09(LIG) A|401:O10(LIG)

 Have added a total of        10 BCORrels for bonds which do not already have a BCORrel


 No bonds (for potential BCORrel addition) were skipped due to both atoms having constant/fixed B(iso)


 ----------- Deactivate hydrogen-free planes if hydrogen atoms present

 A total of       0 planes have been deactivated because they are duplicated hydrogen-free and with-hydrogen forms.

 ----------- Handle peptide omega angle(s)

      Torsion angles split into peptide omega and other
         Number of peptide omega angles is       698
         Number of other torsion is             1266
      Peptide omega angles are monitored rather than directly restrained (PLANE used instead).

 ----------- Fix cis-PRO

 Now apply the fix to get correct restraints for cis PRO residues
   Have got      11 bonds records and      26 angles
   from GEOMETRY cards for the special PROC residue
   Weight for ideal-distance contact term=     4.000

 ----------- Prepare SSBOND/LINK for output PDB file


 ----------- Prepare for QM


 ----------- Prepare symmetry in utility distance restraints


 ############### Setting up Rhofit contacts (if needed)

 No ligand-(fixed protein) contact term will be used

 ############### Working out initial function and gradient


   MaxLik Scaling Cycle 0002
 
 Initial functional value = 5.199910E+05 rms gradient=      21.1314
 X-ray contribution to function value = 4.879449E+05
 Geometry contribution (inc ncs)      =      32046.1
 Geometry function summary
     term                           number     weight       rms        GooF      function     fn/numb
     ================================================================================================
     BOND bond lengths (angs)       5642       2.000      0.0071       0.353      1405.85       0.249
     ANGL bond angles (degs)        7656       2.000        0.96       0.570      4980.58       0.651
     TORS split-harmonic tors       1266       0.000       16.97       1.068         0.00       0.000
     SINTOR sinusoidal tors         1266       2.000       16.97                  2208.45       1.744
     PLAN general plane (angs)       958       5.000      0.0141       0.706      2388.86       2.494
     BCORrelations (angs^2)         5642      10.000       0.915       0.326      6013.30       1.066
     CONT (bad contacts)               0       5.000                                 0.00
     IDEAL(ideal-dist contact)      3891       4.000                              7882.37
     CHIRAL (gelly semiharmon)       742       5.000                                44.52
     SIM similarity restraint for NCS and/or TARGET                               7122.13
     OCCSUM restraint on sum of occupanies                                           0.00       as none defined
     DISTAN utility restraints interatomic distance                                  0.00       as none defined
     UTILANGLE utility angle restraints                                              0.00       as none defined
     UTILTOR utility restraints on torsion angles                                    0.00       as none defined
     Peptide omega torsion angles                           2.86 degs - monitor only
 
 WARNING: Have |delta/sigma| deviations > 5.0 sigma. Number of outliers for each term: 
 WARNING:         2 bond angles.  Worst is     5.6 sigs   104.80 degs A|27:CA(ILE)=A|27:C(ILE)=A|28:N.B(ASN)

 Weighted rms      0.0071         0.957  (temporary rms bond and angle for BUSTER to pick up!)
 Weighted intra-residual rms(bond) for StdProtein (excluding H)      0.0068

 ############### Running initial check


 Initial Geometry Sanity Check

 Check for duplicate bonds: no duplicates found
 
 Check for duplicate or missing bond angles: all are OK
 Check for bond between an atom and itself not involving symmetry:  all are OK
   There are no bad contacts where Delta/sigma is greater than     6.5
   Worst contact dist=   2.960 std=   3.549 sigma=   0.191 abs(Delta/sigma)=    3.085 ats A|285:O (THR) A|401:C01 (LIG)
   It is likely that the geometry libraries, sequence info and links match the input molecule
 GEOMETRY CHECK OVERALL:  PASS


 ############### Creating geometry/screen report


 ############### Option "tao" - run Tao optimizer

 TAO: Toolkit for Advanced Optimization. Reference: 
     Munson, T., Sarich, J., Wild, S., Benson, S., McInnes, L.C. (2018)
     "TAO User Manual (Revision 3.9)", Mathematics and Computer Science
     Division, Argonne National Laboratory
     ANL/MCS-TM-322, http://www.mcs.anl.gov/petsc/
 
 Have found -keeppdb flag so will write out a .pdb file every 1000000 its during opt
 Will apply TAO optimization 
 Maximum number of iterations      100
 Convergence test on Grms       4.0000 (-glim)
 Limit on rms displacement to original position (rmsD2init): NONE test inactive (-dlim)

    analysing argument no 0 : -autoncs with j=0
    analysing argument no 1 : -hydrogens_input_occ2zero with j=0
    analysing argument no 2 : -hydrogens_output_occ2heavy with j=0
    analysing argument no 3 : -screen with j=0
    analysing argument no 4 : 100 with j=0
    analysing argument no 5 : -screen_sigma with j=0
    analysing argument no 6 : 3.0 with j=0
    analysing argument no 7 : -keeppdb with j=0
    analysing argument no 8 : 1000000 with j=0
    analysing argument no 9 : -taomonitor_verbose with j=0
    found argument starting with "-tao" : -taomonitor_verbose
    analysing argument no 10 : 2 with j=1
    found value after -tao/-options_/-viewer_ argument : "2"
 PETSC/TAO: setting option "-taomonitor_verbose" = "2"


   MaxLik Scaling Cycle 0003
   Iters     Tot_Funct    Xray_Funct    Geom_Funct          Grms     rmsD2init     maxD2init             R         Rfree           LLG       LLGfree     rms(bond)    rms(angle)       Opttag_H
   Iters             K   B_IMPF_FRAG        K_MISS   B_TEMP_MISS   K_IMPF_MISS   B_IMPF_MISS        K_SOLV   B_TEMP_SOLV   K_IMPF_SOLV   B_IMPF_SOLV        B_TEMP     B_TEMP_11     B_TEMP_12     B_TEMP_22     B_TEMP_13     B_TEMP_23     B_TEMP_33      K_WILSON      B_WILSON      FGCYCLES       Scatag_H
   Iters          BOND         ANGLE      IMPROPER       TORSION    SINTORSION      TRIGONAL        PLANAR  BCORRELATION       CONTACT     IDEALDIST        CHIRAL    SIMILARITY       OCCSSUM  UTILDISTANCE     UTILANGLE   UTILTORSION        RHOFIT        ADP-01        ADP-02        ADP-03        ADP-04        ADP-05        ADP-06        ADP-07        ADP-08        ADP-09        ADP-10      RHOFITPC            QM       Funtag_H
       0  5.199910E+05  4.879449E+05       32046.1       21.1314  0.000000E+00  0.000000E+00      0.227674      0.233855  0.000000E+00  0.000000E+00    0.00709136      0.956639       Opttag_D
       0       63.8678       4.20042       1.00000  0.000000E+00       1.00000  0.000000E+00      0.901839       55.3997       1.00000       11.9950  0.000000E+00     -0.484731  0.000000E+00     -0.484731  0.000000E+00  0.000000E+00      0.969462       61.3971     -0.203006        2       Scatag_D
       0       1405.85       4980.58  0.000000E+00  0.000000E+00       2208.45  0.000000E+00       2388.86       6013.30  0.000000E+00       7882.37       44.5233       7122.13 -1.000000E+10 -1.000000E+10 -1.000000E+10 -1.000000E+10 -1.000000E+10  0.000000E+00  0.000000E+00  0.000000E+00  0.000000E+00  0.000000E+00  0.000000E+00  0.000000E+00  0.000000E+00  0.000000E+00  0.000000E+00  0.000000E+00  0.000000E+00       Funtag_D
   Geometry Restraint Deviations SCREEN Information

 

 WARNING: have displacement of 15.34 Angs from initial (> 15.00 Angs)
          for variable number= 6847 (type Z        ; returned function value as   2.026949E+010 )
          Involving atom A|217:O

 An atom has moved more than     1.50 A from the initial/last
 position - if this was an accepted trial point, probably need to update the
 non-bonded contact list.


   MaxLik Scaling Cycle 0004

   MaxLik Scaling Cycle 0005

   MaxLik Scaling Cycle 0006

   Wilson plot

   Statistics in resolution bins after refinement cycle 0001
```


|  |  |
| --- | --- |
|  | - Rxpct factors plot- Rxpct factors table- <Log-Lik Gain>- Ln Sigma A- Correlation Coefficients |


```
   Iters     Tot_Funct    Xray_Funct    Geom_Funct          Grms     rmsD2init     maxD2init             R         Rfree           LLG       LLGfree     rms(bond)    rms(angle)
   Iters             K   B_IMPF_FRAG        K_MISS   B_TEMP_MISS   K_IMPF_MISS   B_IMPF_MISS        K_SOLV   B_TEMP_SOLV   K_IMPF_SOLV   B_IMPF_SOLV        B_TEMP     B_TEMP_11     B_TEMP_12     B_TEMP_22     B_TEMP_13     B_TEMP_23     B_TEMP_33      K_WILSON      B_WILSON      FGCYCLES
   Iters          BOND         ANGLE      IMPROPER       TORSION    SINTORSION      TRIGONAL        PLANAR  BCORRELATION       CONTACT     IDEALDIST        CHIRAL    SIMILARITY       OCCSSUM  UTILDISTANCE     UTILANGLE   UTILTORSION        RHOFIT        ADP-01        ADP-02        ADP-03        ADP-04        ADP-05        ADP-06        ADP-07        ADP-08        ADP-09        ADP-10      RHOFITPC            QM
       1  5.198865E+05  4.876588E+05       32227.7       23.9252  5.949288E-04    0.00411763      0.227509      0.233854  6.319932E-04  6.993745E-05    0.00716182      0.958396       Opttag_D
       1       63.8599       4.18489       1.00000  0.000000E+00       1.00000  0.000000E+00      0.901641       55.3585       1.00000       12.0147  0.000000E+00     -0.490279  0.000000E+00     -0.490279  0.000000E+00  0.000000E+00      0.980557       61.3971     -0.203006        3       Scatag_D
       1       1433.92       4998.89  0.000000E+00  0.000000E+00       2208.52  0.000000E+00       2394.73       6153.57  0.000000E+00       7881.24       44.8492       7112.00 -1.000000E+10 -1.000000E+10 -1.000000E+10 -1.000000E+10 -1.000000E+10  0.000000E+00  0.000000E+00  0.000000E+00  0.000000E+00  0.000000E+00  0.000000E+00  0.000000E+00  0.000000E+00  0.000000E+00  0.000000E+00  0.000000E+00  0.000000E+00       Funtag_D
   Geometry Restraint Deviations SCREEN Information

 

   MaxLik Scaling Cycle 0007

   Wilson plot

   Statistics in resolution bins after refinement cycle 0002
```


|  |  |
| --- | --- |
|  | - Rxpct factors plot- Rxpct factors table- <Log-Lik Gain>- Ln Sigma A- Correlation Coefficients |


```
   Iters     Tot_Funct    Xray_Funct    Geom_Funct          Grms     rmsD2init     maxD2init             R         Rfree           LLG       LLGfree     rms(bond)    rms(angle)
   Iters             K   B_IMPF_FRAG        K_MISS   B_TEMP_MISS   K_IMPF_MISS   B_IMPF_MISS        K_SOLV   B_TEMP_SOLV   K_IMPF_SOLV   B_IMPF_SOLV        B_TEMP     B_TEMP_11     B_TEMP_12     B_TEMP_22     B_TEMP_13     B_TEMP_23     B_TEMP_33      K_WILSON      B_WILSON      FGCYCLES
   Iters          BOND         ANGLE      IMPROPER       TORSION    SINTORSION      TRIGONAL        PLANAR  BCORRELATION       CONTACT     IDEALDIST        CHIRAL    SIMILARITY       OCCSSUM  UTILDISTANCE     UTILANGLE   UTILTORSION        RHOFIT        ADP-01        ADP-02        ADP-03        ADP-04        ADP-05        ADP-06        ADP-07        ADP-08        ADP-09        ADP-10      RHOFITPC            QM
       2  5.197898E+05  4.872175E+05       32572.3       36.6035    0.00201187     0.0158956      0.227198      0.233817    0.00160988  1.234793E-04    0.00735941      0.963187       Opttag_D
       2       63.8606       4.16576       1.00000  0.000000E+00       1.00000  0.000000E+00      0.901557       55.3455       1.00000       11.9983  0.000000E+00     -0.502907  0.000000E+00     -0.502907  0.000000E+00  0.000000E+00       1.00581       61.3971     -0.203006        1       Scatag_D
       2       1514.13       5049.00  0.000000E+00  0.000000E+00       2208.87  0.000000E+00       2414.54       6349.97  0.000000E+00       7882.41       45.0101       7108.41 -1.000000E+10 -1.000000E+10 -1.000000E+10 -1.000000E+10 -1.000000E+10  0.000000E+00  0.000000E+00  0.000000E+00  0.000000E+00  0.000000E+00  0.000000E+00  0.000000E+00  0.000000E+00  0.000000E+00  0.000000E+00  0.000000E+00  0.000000E+00       Funtag_D
   Geometry Restraint Deviations SCREEN Information

 
       3  5.196908E+05  4.870653E+05       32625.5       27.1390    0.00258102     0.0210704      0.227090      0.233795    0.00194693  1.445716E-04    0.00741462      0.964032       Opttag_D
       3       63.8616       4.15972       1.00000  0.000000E+00       1.00000  0.000000E+00      0.901552       55.3440       1.00000       11.9888  0.000000E+00     -0.507093  0.000000E+00     -0.507093  0.000000E+00  0.000000E+00       1.01419       61.3971     -0.203006        1       Scatag_D
       3       1536.94       5057.85  0.000000E+00  0.000000E+00       2209.11  0.000000E+00       2423.73       6356.31  0.000000E+00       7885.13       45.0810       7111.32 -1.000000E+10 -1.000000E+10 -1.000000E+10 -1.000000E+10 -1.000000E+10  0.000000E+00  0.000000E+00  0.000000E+00  0.000000E+00  0.000000E+00  0.000000E+00  0.000000E+00  0.000000E+00  0.000000E+00  0.000000E+00  0.000000E+00  0.000000E+00       Funtag_D
       4  5.196191E+05  4.868607E+05       32758.4       24.5623    0.00337880     0.0282355      0.226948      0.233761    0.00240034  1.817178E-04    0.00748205      0.966014       Opttag_D
       4       63.8631       4.15156       1.00000  0.000000E+00       1.00000  0.000000E+00      0.901545       55.3411       1.00000       11.9766  0.000000E+00     -0.512387  0.000000E+00     -0.512387  0.000000E+00  0.000000E+00       1.02477       61.3971     -0.203006        1       Scatag_D
       4       1565.02       5078.68  0.000000E+00  0.000000E+00       2209.49  0.000000E+00       2436.77       6415.24  0.000000E+00       7889.47       45.2715       7118.48 -1.000000E+10 -1.000000E+10 -1.000000E+10 -1.000000E+10 -1.000000E+10  0.000000E+00  0.000000E+00  0.000000E+00  0.000000E+00  0.000000E+00  0.000000E+00  0.000000E+00  0.000000E+00  0.000000E+00  0.000000E+00  0.000000E+00  0.000000E+00       Funtag_D
       5  5.195409E+05  4.864985E+05       33042.4       26.2942    0.00486362     0.0412966      0.226696      0.233697    0.00320281  2.591699E-04    0.00760540      0.970777       Opttag_D
       5       63.8666       4.13734       1.00000  0.000000E+00       1.00000  0.000000E+00      0.901536       55.3372       1.00000       11.9544  0.000000E+00     -0.521919  0.000000E+00     -0.521919  0.000000E+00  0.000000E+00       1.04384       61.3971     -0.203006        1       Scatag_D
       5       1617.05       5128.88  0.000000E+00  0.000000E+00       2210.24  0.000000E+00       2461.54       6538.74  0.000000E+00       7899.65       45.7124       7140.60 -1.000000E+10 -1.000000E+10 -1.000000E+10 -1.000000E+10 -1.000000E+10  0.000000E+00  0.000000E+00  0.000000E+00  0.000000E+00  0.000000E+00  0.000000E+00  0.000000E+00  0.000000E+00  0.000000E+00  0.000000E+00  0.000000E+00  0.000000E+00       Funtag_D
       6  5.194610E+05  4.861451E+05       33316.0       22.8606    0.00646604     0.0551327      0.226446      0.233616    0.00398581  3.884560E-04    0.00770445      0.974949       Opttag_D
       6       63.8723       4.12432       1.00000  0.000000E+00       1.00000  0.000000E+00      0.901558       55.3383       1.00000       11.9279  0.000000E+00     -0.531583  0.000000E+00     -0.531583  0.000000E+00  0.000000E+00       1.06317       61.3971     -0.203006        1       Scatag_D
       6       1659.44       5173.06  0.000000E+00  0.000000E+00       2211.20  0.000000E+00       2488.47       6656.28  0.000000E+00       7913.16       46.2088       7168.14 -1.000000E+10 -1.000000E+10 -1.000000E+10 -1.000000E+10 -1.000000E+10  0.000000E+00  0.000000E+00  0.000000E+00  0.000000E+00  0.000000E+00  0.000000E+00  0.000000E+00  0.000000E+00  0.000000E+00  0.000000E+00  0.000000E+00  0.000000E+00       Funtag_D
       7  5.193824E+05  4.859085E+05       33473.9       19.9111    0.00782147     0.0673599      0.226271      0.233523    0.00451000  5.205601E-04    0.00772359      0.978256       Opttag_D
       7       63.8800       4.11686       1.00000  0.000000E+00       1.00000  0.000000E+00      0.901622       55.3468       1.00000       11.9019  0.000000E+00     -0.538316  0.000000E+00     -0.538316  0.000000E+00  0.000000E+00       1.07663       61.3971     -0.203006        1       Scatag_D
       7       1667.70       5208.21  0.000000E+00  0.000000E+00       2212.18  0.000000E+00       2508.90       6722.30  0.000000E+00       7927.10       46.6216       7180.93 -1.000000E+10 -1.000000E+10 -1.000000E+10 -1.000000E+10 -1.000000E+10  0.000000E+00  0.000000E+00  0.000000E+00  0.000000E+00  0.000000E+00  0.000000E+00  0.000000E+00  0.000000E+00  0.000000E+00  0.000000E+00  0.000000E+00  0.000000E+00       Funtag_D
       8  5.193114E+05  4.857469E+05       33564.5       19.3703    0.00909469     0.0817967      0.226140      0.233429    0.00486807  6.274730E-04    0.00770199      0.980916       Opttag_D
       8       63.8893       4.11309       1.00000  0.000000E+00       1.00000  0.000000E+00      0.901712       55.3622       1.00000       11.8750  0.000000E+00     -0.543258  0.000000E+00     -0.543258  0.000000E+00  0.000000E+00       1.08652       61.3971     -0.203006        1       Scatag_D
       8       1658.38       5236.57  0.000000E+00  0.000000E+00       2213.16  0.000000E+00       2524.12       6761.20  0.000000E+00       7942.09       47.0135       7182.01 -1.000000E+10 -1.000000E+10 -1.000000E+10 -1.000000E+10 -1.000000E+10  0.000000E+00  0.000000E+00  0.000000E+00  0.000000E+00  0.000000E+00  0.000000E+00  0.000000E+00  0.000000E+00  0.000000E+00  0.000000E+00  0.000000E+00  0.000000E+00       Funtag_D
       9  5.192477E+05  4.856032E+05       33644.6       22.4052     0.0105856     0.0987428      0.226013      0.233318    0.00518646  7.301163E-04    0.00766605      0.984181       Opttag_D
       9       63.9012       4.11083       1.00000  0.000000E+00       1.00000  0.000000E+00      0.901827       55.3842       1.00000       11.8429  0.000000E+00     -0.547751  0.000000E+00     -0.547751  0.000000E+00  0.000000E+00       1.09550       61.3971     -0.203006        1       Scatag_D
       9       1642.94       5271.50  0.000000E+00  0.000000E+00       2214.50  0.000000E+00       2537.70       6788.57  0.000000E+00       7961.19       47.4447       7180.74 -1.000000E+10 -1.000000E+10 -1.000000E+10 -1.000000E+10 -1.000000E+10  0.000000E+00  0.000000E+00  0.000000E+00  0.000000E+00  0.000000E+00  0.000000E+00  0.000000E+00  0.000000E+00  0.000000E+00  0.000000E+00  0.000000E+00  0.000000E+00       Funtag_D
      10  5.191828E+05  4.855027E+05       33680.1       22.5846     0.0121306      0.116129      0.225912      0.233221    0.00540899  7.723861E-04    0.00761323      0.986824       Opttag_D
      10       63.9140       4.11049       1.00000  0.000000E+00       1.00000  0.000000E+00      0.901950       55.4089       1.00000       11.8104  0.000000E+00     -0.550540  0.000000E+00     -0.550540  0.000000E+00  0.000000E+00       1.10108       61.3971     -0.203006        1       Scatag_D
      10       1620.38       5299.85  0.000000E+00  0.000000E+00       2215.81  0.000000E+00       2546.36       6790.16  0.000000E+00       7982.64       47.8138       7177.11 -1.000000E+10 -1.000000E+10 -1.000000E+10 -1.000000E+10 -1.000000E+10  0.000000E+00  0.000000E+00  0.000000E+00  0.000000E+00  0.000000E+00  0.000000E+00  0.000000E+00  0.000000E+00  0.000000E+00  0.000000E+00  0.000000E+00  0.000000E+00       Funtag_D
      11  5.191263E+05  4.854375E+05       33688.7       21.0423     0.0131173      0.126751      0.225848      0.233179    0.00555337  7.636760E-04    0.00759194      0.987742       Opttag_D
      11       63.9216       4.10989       1.00000  0.000000E+00       1.00000  0.000000E+00      0.902003       55.4244       1.00000       11.7908  0.000000E+00     -0.551968  0.000000E+00     -0.551968  0.000000E+00  0.000000E+00       1.10394       61.3971     -0.203006        1       Scatag_D
      11       1611.33       5309.70  0.000000E+00  0.000000E+00       2216.70  0.000000E+00       2548.35       6783.91  0.000000E+00       7996.60       47.9529       7174.18 -1.000000E+10 -1.000000E+10 -1.000000E+10 -1.000000E+10 -1.000000E+10  0.000000E+00  0.000000E+00  0.000000E+00  0.000000E+00  0.000000E+00  0.000000E+00  0.000000E+00  0.000000E+00  0.000000E+00  0.000000E+00  0.000000E+00  0.000000E+00       Funtag_D
      12  5.190644E+05  4.853214E+05       33743.0       21.3456     0.0145594      0.141001      0.225742      0.233137    0.00581078  7.661524E-04    0.00759736      0.989350       Opttag_D
      12       63.9321       4.10782       1.00000  0.000000E+00       1.00000  0.000000E+00      0.902061       55.4441       1.00000       11.7638  0.000000E+00     -0.554637  0.000000E+00     -0.554637  0.000000E+00  0.000000E+00       1.10927       61.3971     -0.203006        1       Scatag_D
      12       1613.63       5327.01  0.000000E+00  0.000000E+00       2217.93  0.000000E+00       2551.76       6791.78  0.000000E+00       8016.42       48.1812       7176.33 -1.000000E+10 -1.000000E+10 -1.000000E+10 -1.000000E+10 -1.000000E+10  0.000000E+00  0.000000E+00  0.000000E+00  0.000000E+00  0.000000E+00  0.000000E+00  0.000000E+00  0.000000E+00  0.000000E+00  0.000000E+00  0.000000E+00  0.000000E+00       Funtag_D
      13  5.190189E+05  4.851437E+05       33875.2       25.5649     0.0164873      0.158427      0.225591      0.233092    0.00620439  7.955278E-04    0.00762669      0.992339       Opttag_D
      13       63.9457       4.10386       1.00000  0.000000E+00       1.00000  0.000000E+00      0.902125       55.4659       1.00000       11.7312  0.000000E+00     -0.559023  0.000000E+00     -0.559023  0.000000E+00  0.000000E+00       1.11805       61.3971     -0.203006        1       Scatag_D
      13       1626.11       5359.24  0.000000E+00  0.000000E+00       2219.50  0.000000E+00       2560.31       6830.38  0.000000E+00       8041.89       48.6379       7189.12 -1.000000E+10 -1.000000E+10 -1.000000E+10 -1.000000E+10 -1.000000E+10  0.000000E+00  0.000000E+00  0.000000E+00  0.000000E+00  0.000000E+00  0.000000E+00  0.000000E+00  0.000000E+00  0.000000E+00  0.000000E+00  0.000000E+00  0.000000E+00       Funtag_D
      14  5.189698E+05  4.850379E+05       33931.8       22.1457     0.0174174      0.165729      0.225508      0.233082    0.00643872  8.219999E-04    0.00765250      0.993246       Opttag_D
      14       63.9520       4.10095       1.00000  0.000000E+00       1.00000  0.000000E+00      0.902138       55.4747       1.00000       11.7173  0.000000E+00     -0.561773  0.000000E+00     -0.561773  0.000000E+00  0.000000E+00       1.12355       61.3971     -0.203006        1       Scatag_D
      14       1637.14       5369.05  0.000000E+00  0.000000E+00       2220.15  0.000000E+00       2565.18       6841.65  0.000000E+00       8053.14       48.9543       7196.58 -1.000000E+10 -1.000000E+10 -1.000000E+10 -1.000000E+10 -1.000000E+10  0.000000E+00  0.000000E+00  0.000000E+00  0.000000E+00  0.000000E+00  0.000000E+00  0.000000E+00  0.000000E+00  0.000000E+00  0.000000E+00  0.000000E+00  0.000000E+00       Funtag_D
      15  5.189167E+05  4.848666E+05       34050.1       22.5322     0.0189671      0.177095      0.225376      0.233056    0.00681839  8.754563E-04    0.00768797      0.995213       Opttag_D
      15       63.9631       4.09646       1.00000  0.000000E+00       1.00000  0.000000E+00      0.902170       55.4887       1.00000       11.6953  0.000000E+00     -0.566310  0.000000E+00     -0.566310  0.000000E+00  0.000000E+00       1.13262       61.3971     -0.203006        1       Scatag_D
      15       1652.35       5390.33  0.000000E+00  0.000000E+00       2221.26  0.000000E+00       2575.09       6880.60  0.000000E+00       8071.61       49.6196       7209.29 -1.000000E+10 -1.000000E+10 -1.000000E+10 -1.000000E+10 -1.000000E+10  0.000000E+00  0.000000E+00  0.000000E+00  0.000000E+00  0.000000E+00  0.000000E+00  0.000000E+00  0.000000E+00  0.000000E+00  0.000000E+00  0.000000E+00  0.000000E+00       Funtag_D
      16  5.188716E+05  4.846690E+05       34202.7       25.9252     0.0210998      0.191426      0.225214      0.233015    0.00725623  9.036363E-04    0.00772174      0.998039       Opttag_D
      16       63.9796       4.09216       1.00000  0.000000E+00       1.00000  0.000000E+00      0.902234       55.5105       1.00000       11.6646  0.000000E+00     -0.571240  0.000000E+00     -0.571240  0.000000E+00  0.000000E+00       1.14248       61.3971     -0.203006        1       Scatag_D
      16       1666.90       5420.99  0.000000E+00  0.000000E+00       2222.88  0.000000E+00       2587.82       6928.94  0.000000E+00       8098.45       50.5158       7226.19 -1.000000E+10 -1.000000E+10 -1.000000E+10 -1.000000E+10 -1.000000E+10  0.000000E+00  0.000000E+00  0.000000E+00  0.000000E+00  0.000000E+00  0.000000E+00  0.000000E+00  0.000000E+00  0.000000E+00  0.000000E+00  0.000000E+00  0.000000E+00       Funtag_D
      17  5.188237E+05  4.844733E+05       34350.4       25.4919     0.0234857      0.205866      0.225046      0.232969    0.00768973  9.020992E-04    0.00775667       1.00112       Opttag_D
      17       63.9989       4.08854       1.00000  0.000000E+00       1.00000  0.000000E+00      0.902310       55.5371       1.00000       11.6312  0.000000E+00     -0.576127  0.000000E+00     -0.576127  0.000000E+00  0.000000E+00       1.15225       61.3971     -0.203006        1       Scatag_D
      17       1682.01       5454.56  0.000000E+00  0.000000E+00       2224.89  0.000000E+00       2602.02       6959.29  0.000000E+00       8129.87       51.4505       7246.30 -1.000000E+10 -1.000000E+10 -1.000000E+10 -1.000000E+10 -1.000000E+10  0.000000E+00  0.000000E+00  0.000000E+00  0.000000E+00  0.000000E+00  0.000000E+00  0.000000E+00  0.000000E+00  0.000000E+00  0.000000E+00  0.000000E+00  0.000000E+00       Funtag_D
      18  5.187847E+05  4.843834E+05       34401.3       25.3595     0.0248952      0.212133      0.224961      0.232933    0.00788889  8.782743E-04    0.00775550       1.00238       Opttag_D
      18       64.0117       4.08777       1.00000  0.000000E+00       1.00000  0.000000E+00      0.902367       55.5558       1.00000       11.6108  0.000000E+00     -0.578167  0.000000E+00     -0.578167  0.000000E+00  0.000000E+00       1.15633       61.3971     -0.203006        1       Scatag_D
      18       1681.50       5468.25  0.000000E+00  0.000000E+00       2226.26  0.000000E+00       2609.04       6962.32  0.000000E+00       8148.72       51.7697       7253.41 -1.000000E+10 -1.000000E+10 -1.000000E+10 -1.000000E+10 -1.000000E+10  0.000000E+00  0.000000E+00  0.000000E+00  0.000000E+00  0.000000E+00  0.000000E+00  0.000000E+00  0.000000E+00  0.000000E+00  0.000000E+00  0.000000E+00  0.000000E+00       Funtag_D
      19  5.187411E+05  4.842826E+05       34458.6       26.8534     0.0267462      0.217865      0.224860      0.232893    0.00811231  8.448000E-04    0.00775047       1.00400       Opttag_D
      19       64.0289       4.08755       1.00000  0.000000E+00       1.00000  0.000000E+00      0.902435       55.5826       1.00000       11.5847  0.000000E+00     -0.580241  0.000000E+00     -0.580241  0.000000E+00  0.000000E+00       1.16048       61.3971     -0.203006        1       Scatag_D
      19       1679.33       5485.99  0.000000E+00  0.000000E+00       2228.25  0.000000E+00       2617.35       6959.50  0.000000E+00       8173.81       52.0108       7262.32 -1.000000E+10 -1.000000E+10 -1.000000E+10 -1.000000E+10 -1.000000E+10  0.000000E+00  0.000000E+00  0.000000E+00  0.000000E+00  0.000000E+00  0.000000E+00  0.000000E+00  0.000000E+00  0.000000E+00  0.000000E+00  0.000000E+00  0.000000E+00       Funtag_D
      20  5.187058E+05  4.841930E+05       34512.8       28.0598     0.0285074      0.222893      0.224772      0.232866    0.00831074  8.109841E-04    0.00775131       1.00532       Opttag_D
      20       64.0451       4.08745       1.00000  0.000000E+00       1.00000  0.000000E+00      0.902478       55.6084       1.00000       11.5617  0.000000E+00     -0.582012  0.000000E+00     -0.582012  0.000000E+00  0.000000E+00       1.16402       61.3971     -0.203006        1       Scatag_D
      20       1679.69       5500.40  0.000000E+00  0.000000E+00       2230.32  0.000000E+00       2625.15       6954.65  0.000000E+00       8197.67       52.2234       7272.67 -1.000000E+10 -1.000000E+10 -1.000000E+10 -1.000000E+10 -1.000000E+10  0.000000E+00  0.000000E+00  0.000000E+00  0.000000E+00  0.000000E+00  0.000000E+00  0.000000E+00  0.000000E+00  0.000000E+00  0.000000E+00  0.000000E+00  0.000000E+00       Funtag_D
      21  5.186671E+05  4.841381E+05       34529.0       26.3621     0.0296157      0.233677      0.224721      0.232854    0.00843240  8.157662E-04    0.00774743       1.00566       Opttag_D
      21       64.0552       4.08735       1.00000  0.000000E+00       1.00000  0.000000E+00      0.902486       55.6246       1.00000       11.5489  0.000000E+00     -0.582966  0.000000E+00     -0.582966  0.000000E+00  0.000000E+00       1.16593       61.3971     -0.203006        1       Scatag_D
      21       1678.01       5504.12  0.000000E+00  0.000000E+00       2231.76  0.000000E+00       2629.60       6943.07  0.000000E+00       8211.81       52.3092       7278.31 -1.000000E+10 -1.000000E+10 -1.000000E+10 -1.000000E+10 -1.000000E+10  0.000000E+00  0.000000E+00  0.000000E+00  0.000000E+00  0.000000E+00  0.000000E+00  0.000000E+00  0.000000E+00  0.000000E+00  0.000000E+00  0.000000E+00  0.000000E+00       Funtag_D
      22  5.186186E+05  4.840152E+05       34603.4       26.7071     0.0319009      0.255426      0.224610      0.232837    0.00870463  8.272943E-04    0.00775798       1.00694       Opttag_D
      22       64.0754       4.08651       1.00000  0.000000E+00       1.00000  0.000000E+00      0.902491       55.6555       1.00000       11.5247  0.000000E+00     -0.585381  0.000000E+00     -0.585381  0.000000E+00  0.000000E+00       1.17076       61.3971     -0.203006        1       Scatag_D
      22       1682.58       5518.11  0.000000E+00  0.000000E+00       2234.66  0.000000E+00       2639.90       6941.91  0.000000E+00       8239.76       52.6957       7293.81 -1.000000E+10 -1.000000E+10 -1.000000E+10 -1.000000E+10 -1.000000E+10  0.000000E+00  0.000000E+00  0.000000E+00  0.000000E+00  0.000000E+00  0.000000E+00  0.000000E+00  0.000000E+00  0.000000E+00  0.000000E+00  0.000000E+00  0.000000E+00       Funtag_D
      23  5.185880E+05  4.838963E+05       34691.7       28.9213     0.0343257      0.278181      0.224502      0.232831    0.00896809  7.991997E-04    0.00777572       1.00838       Opttag_D
      23       64.0977       4.08622       1.00000  0.000000E+00       1.00000  0.000000E+00      0.902503       55.6893       1.00000       11.5003  0.000000E+00     -0.588084  0.000000E+00     -0.588084  0.000000E+00  0.000000E+00       1.17617       61.3971     -0.203006        1       Scatag_D
      23       1690.28       5533.86  0.000000E+00  0.000000E+00       2237.71  0.000000E+00       2650.83       6945.57  0.000000E+00       8268.59       53.1049       7311.74 -1.000000E+10 -1.000000E+10 -1.000000E+10 -1.000000E+10 -1.000000E+10  0.000000E+00  0.000000E+00  0.000000E+00  0.000000E+00  0.000000E+00  0.000000E+00  0.000000E+00  0.000000E+00  0.000000E+00  0.000000E+00  0.000000E+00  0.000000E+00       Funtag_D
      24  5.185555E+05  4.838620E+05       34693.5       25.8171     0.0350003      0.284639      0.224470      0.232830    0.00904404  7.927952E-04    0.00777515       1.00855       Opttag_D
      24       64.1046       4.08620       1.00000  0.000000E+00       1.00000  0.000000E+00      0.902508       55.6987       1.00000       11.4939  0.000000E+00     -0.588996  0.000000E+00     -0.588996  0.000000E+00  0.000000E+00       1.17799       61.3971     -0.203006        1       Scatag_D
      24       1690.03       5535.75  0.000000E+00  0.000000E+00       2238.57  0.000000E+00       2653.27       6932.79  0.000000E+00       8275.42       53.2040       7314.45 -1.000000E+10 -1.000000E+10 -1.000000E+10 -1.000000E+10 -1.000000E+10  0.000000E+00  0.000000E+00  0.000000E+00  0.000000E+00  0.000000E+00  0.000000E+00  0.000000E+00  0.000000E+00  0.000000E+00  0.000000E+00  0.000000E+00  0.000000E+00       Funtag_D
      25  5.185170E+05  4.837879E+05       34729.1       24.8456     0.0366255      0.300026      0.224400      0.232830    0.00920833  7.579546E-04    0.00778065       1.00932       Opttag_D
      25       64.1217       4.08662       1.00000  0.000000E+00       1.00000  0.000000E+00      0.902529       55.7224       1.00000       11.4781  0.000000E+00     -0.591150  0.000000E+00     -0.591150  0.000000E+00  0.000000E+00       1.18230       61.3971     -0.203006        1       Scatag_D
      25       1692.43       5544.21  0.000000E+00  0.000000E+00       2240.67  0.000000E+00       2659.24       6924.31  0.000000E+00       8292.63       53.4128       7322.23 -1.000000E+10 -1.000000E+10 -1.000000E+10 -1.000000E+10 -1.000000E+10  0.000000E+00  0.000000E+00  0.000000E+00  0.000000E+00  0.000000E+00  0.000000E+00  0.000000E+00  0.000000E+00  0.000000E+00  0.000000E+00  0.000000E+00  0.000000E+00       Funtag_D
      26  5.184869E+05  4.837125E+05       34774.4       24.6619     0.0385192      0.317807      0.224328      0.232832    0.00937527  7.114150E-04    0.00778829       1.01014       Opttag_D
      26       64.1413       4.08752       1.00000  0.000000E+00       1.00000  0.000000E+00      0.902554       55.7498       1.00000       11.4607  0.000000E+00     -0.593459  0.000000E+00     -0.593459  0.000000E+00  0.000000E+00       1.18692       61.3971     -0.203006        1       Scatag_D
      26       1695.75       5553.27  0.000000E+00  0.000000E+00       2243.32  0.000000E+00       2666.22       6916.54  0.000000E+00       8313.41       53.6249       7332.21 -1.000000E+10 -1.000000E+10 -1.000000E+10 -1.000000E+10 -1.000000E+10  0.000000E+00  0.000000E+00  0.000000E+00  0.000000E+00  0.000000E+00  0.000000E+00  0.000000E+00  0.000000E+00  0.000000E+00  0.000000E+00  0.000000E+00  0.000000E+00       Funtag_D
      27  5.184587E+05  4.835569E+05       34901.7       28.4645     0.0430925      0.360208      0.224167      0.232843    0.00971998  5.934863E-04    0.00780885       1.01236       Opttag_D
      27       64.1892       4.09077       1.00000  0.000000E+00       1.00000  0.000000E+00      0.902616       55.8141       1.00000       11.4224  0.000000E+00     -0.598982  0.000000E+00     -0.598982  0.000000E+00  0.000000E+00       1.19796       61.3971     -0.203006        1       Scatag_D
      27       1704.72       5577.72  0.000000E+00  0.000000E+00       2250.03  0.000000E+00       2682.80       6911.99  0.000000E+00       8363.38       54.2047       7356.88 -1.000000E+10 -1.000000E+10 -1.000000E+10 -1.000000E+10 -1.000000E+10  0.000000E+00  0.000000E+00  0.000000E+00  0.000000E+00  0.000000E+00  0.000000E+00  0.000000E+00  0.000000E+00  0.000000E+00  0.000000E+00  0.000000E+00  0.000000E+00       Funtag_D
      28  5.184183E+05  4.835456E+05       34872.7       22.6346     0.0436423      0.365483      0.224151      0.232842    0.00974513  5.773469E-04    0.00779935       1.01200       Opttag_D
      28       64.1952       4.09150       1.00000  0.000000E+00       1.00000  0.000000E+00      0.902620       55.8225       1.00000       11.4185  0.000000E+00     -0.599621  0.000000E+00     -0.599621  0.000000E+00  0.000000E+00       1.19924       61.3971     -0.203006        1       Scatag_D
      28       1700.57       5573.71  0.000000E+00  0.000000E+00       2251.04  0.000000E+00       2683.68       6885.38  0.000000E+00       8368.35       54.2574       7355.73 -1.000000E+10 -1.000000E+10 -1.000000E+10 -1.000000E+10 -1.000000E+10  0.000000E+00  0.000000E+00  0.000000E+00  0.000000E+00  0.000000E+00  0.000000E+00  0.000000E+00  0.000000E+00  0.000000E+00  0.000000E+00  0.000000E+00  0.000000E+00       Funtag_D
      29  5.183992E+05  4.835496E+05       34849.7       21.9325     0.0437076      0.366430      0.224151      0.232838    0.00973635  5.863986E-04    0.00778752       1.01150       Opttag_D
      29       64.1964       4.09188       1.00000  0.000000E+00       1.00000  0.000000E+00      0.902615       55.8239       1.00000       11.4186  0.000000E+00     -0.599800  0.000000E+00     -0.599800  0.000000E+00  0.000000E+00       1.19960       61.3971     -0.203006        1       Scatag_D
      29       1695.42       5568.19  0.000000E+00  0.000000E+00       2251.31  0.000000E+00       2682.45       6879.44  0.000000E+00       8367.48       54.2646       7351.10 -1.000000E+10 -1.000000E+10 -1.000000E+10 -1.000000E+10 -1.000000E+10  0.000000E+00  0.000000E+00  0.000000E+00  0.000000E+00  0.000000E+00  0.000000E+00  0.000000E+00  0.000000E+00  0.000000E+00  0.000000E+00  0.000000E+00  0.000000E+00       Funtag_D
      30  5.183674E+05  4.835261E+05       34841.3       21.4886     0.0449161      0.378110      0.224118      0.232833    0.00978826  5.798233E-04    0.00777457       1.01125       Opttag_D
      30       64.2100       4.09358       1.00000  0.000000E+00       1.00000  0.000000E+00      0.902621       55.8417       1.00000       11.4109  0.000000E+00     -0.601509  0.000000E+00     -0.601509  0.000000E+00  0.000000E+00       1.20302       61.3971     -0.203006        1       Scatag_D
      30       1689.79       5565.42  0.000000E+00  0.000000E+00       2253.52  0.000000E+00       2683.87       6867.78  0.000000E+00       8377.01       54.4100       7349.46 -1.000000E+10 -1.000000E+10 -1.000000E+10 -1.000000E+10 -1.000000E+10  0.000000E+00  0.000000E+00  0.000000E+00  0.000000E+00  0.000000E+00  0.000000E+00  0.000000E+00  0.000000E+00  0.000000E+00  0.000000E+00  0.000000E+00  0.000000E+00       Funtag_D
      31  5.183539E+05  4.834604E+05       34893.5       28.8885     0.0478306      0.411494      0.224033      0.232827    0.00993374  5.344793E-04    0.00776302       1.01167       Opttag_D
      31       64.2424       4.09703       1.00000  0.000000E+00       1.00000  0.000000E+00      0.902645       55.8826       1.00000       11.3932  0.000000E+00     -0.605619  0.000000E+00     -0.605619  0.000000E+00  0.000000E+00       1.21124       61.3971     -0.203006        1       Scatag_D
      31       1684.77       5570.10  0.000000E+00  0.000000E+00       2258.67  0.000000E+00       2689.57       6880.02  0.000000E+00       8401.18       54.5459       7354.60 -1.000000E+10 -1.000000E+10 -1.000000E+10 -1.000000E+10 -1.000000E+10  0.000000E+00  0.000000E+00  0.000000E+00  0.000000E+00  0.000000E+00  0.000000E+00  0.000000E+00  0.000000E+00  0.000000E+00  0.000000E+00  0.000000E+00  0.000000E+00       Funtag_D
      32  5.183127E+05  4.834457E+05       34867.0       19.5145     0.0483798      0.418961      0.224018      0.232830    0.00996648  5.239758E-04    0.00776044       1.01172       Opttag_D
      32       64.2486       4.09761       1.00000  0.000000E+00       1.00000  0.000000E+00      0.902651       55.8906       1.00000       11.3900  0.000000E+00     -0.606455  0.000000E+00     -0.606455  0.000000E+00  0.000000E+00       1.21291       61.3971     -0.203006        1       Scatag_D
      32       1683.65       5570.63  0.000000E+00  0.000000E+00       2259.58  0.000000E+00       2690.88       6845.81  0.000000E+00       8405.96       54.5682       7355.96 -1.000000E+10 -1.000000E+10 -1.000000E+10 -1.000000E+10 -1.000000E+10  0.000000E+00  0.000000E+00  0.000000E+00  0.000000E+00  0.000000E+00  0.000000E+00  0.000000E+00  0.000000E+00  0.000000E+00  0.000000E+00  0.000000E+00  0.000000E+00       Funtag_D
      33  5.182931E+05  4.834155E+05       34877.6       17.6753     0.0493789      0.432545      0.223988      0.232831     0.0100334  5.018588E-04    0.00775781       1.01200       Opttag_D
      33       64.2597       4.09847       1.00000  0.000000E+00       1.00000  0.000000E+00      0.902660       55.9043       1.00000       11.3843  0.000000E+00     -0.608052  0.000000E+00     -0.608052  0.000000E+00  0.000000E+00       1.21610       61.3971     -0.203006        1       Scatag_D
      33       1682.51       5573.76  0.000000E+00  0.000000E+00       2261.26  0.000000E+00       2693.16       6839.86  0.000000E+00       8414.34       54.5463       7358.18 -1.000000E+10 -1.000000E+10 -1.000000E+10 -1.000000E+10 -1.000000E+10  0.000000E+00  0.000000E+00  0.000000E+00  0.000000E+00  0.000000E+00  0.000000E+00  0.000000E+00  0.000000E+00  0.000000E+00  0.000000E+00  0.000000E+00  0.000000E+00       Funtag_D
      34  5.182737E+05  4.833672E+05       34906.5       17.2721     0.0509798      0.455005      0.223937      0.232834     0.0101403  4.629193E-04    0.00776033       1.01252       Opttag_D
      34       64.2778       4.09981       1.00000  0.000000E+00       1.00000  0.000000E+00      0.902673       55.9255       1.00000       11.3764  0.000000E+00     -0.610732  0.000000E+00     -0.610732  0.000000E+00  0.000000E+00       1.22146       61.3971     -0.203006        1       Scatag_D
      34       1683.60       5579.47  0.000000E+00  0.000000E+00       2264.05  0.000000E+00       2696.59       6839.09  0.000000E+00       8427.15       54.5242       7362.02 -1.000000E+10 -1.000000E+10 -1.000000E+10 -1.000000E+10 -1.000000E+10  0.000000E+00  0.000000E+00  0.000000E+00  0.000000E+00  0.000000E+00  0.000000E+00  0.000000E+00  0.000000E+00  0.000000E+00  0.000000E+00  0.000000E+00  0.000000E+00       Funtag_D
      35  5.182453E+05  4.832864E+05       34959.0       16.9219     0.0532235      0.488162      0.223862      0.232841     0.0103194  4.024606E-04    0.00777393       1.01325       Opttag_D
      35       64.3033       4.10105       1.00000  0.000000E+00       1.00000  0.000000E+00      0.902680       55.9515       1.00000       11.3680  0.000000E+00     -0.615004  0.000000E+00     -0.615004  0.000000E+00  0.000000E+00       1.23001       61.3971     -0.203006        1       Scatag_D
      35       1689.51       5587.45  0.000000E+00  0.000000E+00       2268.10  0.000000E+00       2700.77       6848.50  0.000000E+00       8443.30       54.5722       7366.76 -1.000000E+10 -1.000000E+10 -1.000000E+10 -1.000000E+10 -1.000000E+10  0.000000E+00  0.000000E+00  0.000000E+00  0.000000E+00  0.000000E+00  0.000000E+00  0.000000E+00  0.000000E+00  0.000000E+00  0.000000E+00  0.000000E+00  0.000000E+00       Funtag_D
      36  5.182363E+05  4.831689E+05       35067.4       19.9479     0.0570093      0.544234      0.223751      0.232858     0.0105797  2.529362E-04    0.00780746       1.01464       Opttag_D
      36       64.3456       4.10372       1.00000  0.000000E+00       1.00000  0.000000E+00      0.902693       55.9946       1.00000       11.3556  0.000000E+00     -0.621542  0.000000E+00     -0.621542  0.000000E+00  0.000000E+00       1.24308       61.3971     -0.203006        1       Scatag_D
      36       1704.11       5602.87  0.000000E+00  0.000000E+00       2275.23  0.000000E+00       2708.24       6873.29  0.000000E+00       8470.12       54.7016       7378.86 -1.000000E+10 -1.000000E+10 -1.000000E+10 -1.000000E+10 -1.000000E+10  0.000000E+00  0.000000E+00  0.000000E+00  0.000000E+00  0.000000E+00  0.000000E+00  0.000000E+00  0.000000E+00  0.000000E+00  0.000000E+00  0.000000E+00  0.000000E+00       Funtag_D
      37  5.182098E+05  4.831883E+05       35021.5       13.6834     0.0560531      0.532291      0.223772      0.232850     0.0105366  2.899971E-04    0.00779980       1.01410       Opttag_D
      37       64.3353       4.10264       1.00000  0.000000E+00       1.00000  0.000000E+00      0.902681       55.9821       1.00000       11.3596  0.000000E+00     -0.620204  0.000000E+00     -0.620204  0.000000E+00  0.000000E+00       1.24041       61.3971     -0.203006        1       Scatag_D
      37       1700.77       5596.82  0.000000E+00  0.000000E+00       2273.47  0.000000E+00       2705.22       6856.81  0.000000E+00       8460.94       54.6827       7372.79 -1.000000E+10 -1.000000E+10 -1.000000E+10 -1.000000E+10 -1.000000E+10  0.000000E+00  0.000000E+00  0.000000E+00  0.000000E+00  0.000000E+00  0.000000E+00  0.000000E+00  0.000000E+00  0.000000E+00  0.000000E+00  0.000000E+00  0.000000E+00       Funtag_D
      38  5.181993E+05  4.831927E+05       35006.6       13.5530     0.0557236      0.529813      0.223778      0.232847     0.0105270  2.973409E-04    0.00779757       1.01382       Opttag_D
      38       64.3319       4.10217       1.00000  0.000000E+00       1.00000  0.000000E+00      0.902673       55.9770       1.00000       11.3615  0.000000E+00     -0.619779  0.000000E+00     -0.619779  0.000000E+00  0.000000E+00       1.23956       61.3971     -0.203006        1       Scatag_D
      38       1699.80       5593.76  0.000000E+00  0.000000E+00       2272.91  0.000000E+00       2703.44       6856.86  0.000000E+00       8456.12       54.6796       7369.02 -1.000000E+10 -1.000000E+10 -1.000000E+10 -1.000000E+10 -1.000000E+10  0.000000E+00  0.000000E+00  0.000000E+00  0.000000E+00  0.000000E+00  0.000000E+00  0.000000E+00  0.000000E+00  0.000000E+00  0.000000E+00  0.000000E+00  0.000000E+00       Funtag_D
      39  5.181783E+05  4.831805E+05       34997.8       13.9652     0.0559337      0.538793      0.223767      0.232842     0.0105539  2.794936E-04    0.00779900       1.01362       Opttag_D
      39       64.3347       4.10213       1.00000  0.000000E+00       1.00000  0.000000E+00      0.902660       55.9774       1.00000       11.3623  0.000000E+00     -0.620218  0.000000E+00     -0.620218  0.000000E+00  0.000000E+00       1.24044       61.3971     -0.203006        1       Scatag_D
      39       1700.42       5591.61  0.000000E+00  0.000000E+00       2273.46  0.000000E+00       2701.35       6860.20  0.000000E+00       8451.79       54.6570       7364.28 -1.000000E+10 -1.000000E+10 -1.000000E+10 -1.000000E+10 -1.000000E+10  0.000000E+00  0.000000E+00  0.000000E+00  0.000000E+00  0.000000E+00  0.000000E+00  0.000000E+00  0.000000E+00  0.000000E+00  0.000000E+00  0.000000E+00  0.000000E+00       Funtag_D
      40  5.181557E+05  4.831513E+05       35004.4       17.5308     0.0571673      0.567126      0.223735      0.232836     0.0106186  2.356866E-04    0.00779842       1.01353       Opttag_D
      40       64.3483       4.10333       1.00000  0.000000E+00       1.00000  0.000000E+00      0.902646       55.9898       1.00000       11.3605  0.000000E+00     -0.621898  0.000000E+00     -0.621898  0.000000E+00  0.000000E+00       1.24380       61.3971     -0.203006        1       Scatag_D
      40       1700.17       5590.58  0.000000E+00  0.000000E+00       2276.05  0.000000E+00       2699.74       6872.61  0.000000E+00       8450.55       54.4746       7360.22 -1.000000E+10 -1.000000E+10 -1.000000E+10 -1.000000E+10 -1.000000E+10  0.000000E+00  0.000000E+00  0.000000E+00  0.000000E+00  0.000000E+00  0.000000E+00  0.000000E+00  0.000000E+00  0.000000E+00  0.000000E+00  0.000000E+00  0.000000E+00       Funtag_D
      41  5.181290E+05  4.831337E+05       34995.3       12.2983     0.0582149      0.589400      0.223716      0.232840     0.0106576  2.002483E-04    0.00779733       1.01352       Opttag_D
      41       64.3592       4.10458       1.00000  0.000000E+00       1.00000  0.000000E+00      0.902639       56.0009       1.00000       11.3587  0.000000E+00     -0.623097  0.000000E+00     -0.623097  0.000000E+00  0.000000E+00       1.24619       61.3971     -0.203006        1       Scatag_D
      41       1699.69       5590.50  0.000000E+00  0.000000E+00       2278.23  0.000000E+00       2699.17       6863.58  0.000000E+00       8451.09       54.3180       7358.71 -1.000000E+10 -1.000000E+10 -1.000000E+10 -1.000000E+10 -1.000000E+10  0.000000E+00  0.000000E+00  0.000000E+00  0.000000E+00  0.000000E+00  0.000000E+00  0.000000E+00  0.000000E+00  0.000000E+00  0.000000E+00  0.000000E+00  0.000000E+00       Funtag_D
      42  5.181100E+05  4.831217E+05       34988.2       9.91409     0.0591792      0.607859      0.223701      0.232843     0.0106842  1.835111E-04    0.00779074       1.01338       Opttag_D
      42       64.3685       4.10578       1.00000  0.000000E+00       1.00000  0.000000E+00      0.902634       56.0118       1.00000       11.3569  0.000000E+00     -0.624086  0.000000E+00     -0.624086  0.000000E+00  0.000000E+00       1.24817       61.3971     -0.203006        1       Scatag_D
      42       1696.82       5588.93  0.000000E+00  0.000000E+00       2280.18  0.000000E+00       2698.84       6859.95  0.000000E+00       8453.20       54.1325       7356.15 -1.000000E+10 -1.000000E+10 -1.000000E+10 -1.000000E+10 -1.000000E+10  0.000000E+00  0.000000E+00  0.000000E+00  0.000000E+00  0.000000E+00  0.000000E+00  0.000000E+00  0.000000E+00  0.000000E+00  0.000000E+00  0.000000E+00  0.000000E+00       Funtag_D
      43  5.180910E+05  4.831108E+05       34980.2       10.0206     0.0603029      0.630357      0.223682      0.232845     0.0107083  1.659200E-04    0.00778614       1.01309       Opttag_D
      43       64.3786       4.10708       1.00000  0.000000E+00       1.00000  0.000000E+00      0.902622       56.0224       1.00000       11.3560  0.000000E+00     -0.625007  0.000000E+00     -0.625007  0.000000E+00  0.000000E+00       1.25001       61.3971     -0.203006        1       Scatag_D
      43       1694.82       5585.72  0.000000E+00  0.000000E+00       2282.52  0.000000E+00       2697.58       6860.14  0.000000E+00       8454.15       53.8990       7351.34 -1.000000E+10 -1.000000E+10 -1.000000E+10 -1.000000E+10 -1.000000E+10  0.000000E+00  0.000000E+00  0.000000E+00  0.000000E+00  0.000000E+00  0.000000E+00  0.000000E+00  0.000000E+00  0.000000E+00  0.000000E+00  0.000000E+00  0.000000E+00       Funtag_D
      44  5.180752E+05  4.830996E+05       34975.6       11.5686     0.0615969      0.656853      0.223668      0.232851     0.0107332  1.426929E-04    0.00778726       1.01277       Opttag_D
      44       64.3891       4.10834       1.00000  0.000000E+00       1.00000  0.000000E+00      0.902598       56.0317       1.00000       11.3564  0.000000E+00     -0.625957  0.000000E+00     -0.625957  0.000000E+00  0.000000E+00       1.25191       61.3971     -0.203006        1       Scatag_D
      44       1695.31       5582.23  0.000000E+00  0.000000E+00       2285.21  0.000000E+00       2695.38       6864.63  0.000000E+00       8453.85       53.7002       7345.31 -1.000000E+10 -1.000000E+10 -1.000000E+10 -1.000000E+10 -1.000000E+10  0.000000E+00  0.000000E+00  0.000000E+00  0.000000E+00  0.000000E+00  0.000000E+00  0.000000E+00  0.000000E+00  0.000000E+00  0.000000E+00  0.000000E+00  0.000000E+00       Funtag_D
      45  5.180586E+05  4.831002E+05       34958.4       9.16124     0.0619148      0.664750      0.223669      0.232851     0.0107319  1.267243E-04    0.00778821       1.01243       Opttag_D
      45       64.3905       4.10852       1.00000  0.000000E+00       1.00000  0.000000E+00      0.902584       56.0308       1.00000       11.3575  0.000000E+00     -0.625823  0.000000E+00     -0.625823  0.000000E+00  0.000000E+00       1.25165       61.3971     -0.203006        1       Scatag_D
      45       1695.72       5578.48  0.000000E+00  0.000000E+00       2285.89  0.000000E+00       2693.44       6858.89  0.000000E+00       8451.45       53.6994       7340.82 -1.000000E+10 -1.000000E+10 -1.000000E+10 -1.000000E+10 -1.000000E+10  0.000000E+00  0.000000E+00  0.000000E+00  0.000000E+00  0.000000E+00  0.000000E+00  0.000000E+00  0.000000E+00  0.000000E+00  0.000000E+00  0.000000E+00  0.000000E+00       Funtag_D
      46  5.180379E+05  4.831040E+05       34933.9       9.14445     0.0624132      0.678086      0.223669      0.232853     0.0107235  8.283190E-05    0.00779244       1.01192       Opttag_D
      46       64.3922       4.10876       1.00000  0.000000E+00       1.00000  0.000000E+00      0.902557       56.0273       1.00000       11.3600  0.000000E+00     -0.625419  0.000000E+00     -0.625419  0.000000E+00  0.000000E+00       1.25084       61.3971     -0.203006        1       Scatag_D
      46       1697.56       5572.87  0.000000E+00  0.000000E+00       2286.94  0.000000E+00       2689.70       6854.19  0.000000E+00       8446.14       53.7656       7332.76 -1.000000E+10 -1.000000E+10 -1.000000E+10 -1.000000E+10 -1.000000E+10  0.000000E+00  0.000000E+00  0.000000E+00  0.000000E+00  0.000000E+00  0.000000E+00  0.000000E+00  0.000000E+00  0.000000E+00  0.000000E+00  0.000000E+00  0.000000E+00       Funtag_D
      47  5.180207E+05  4.831151E+05       34905.6       10.0978     0.0630137      0.693609      0.223675      0.232856     0.0106989  2.638666E-05    0.00779449       1.01152       Opttag_D
      47       64.3944       4.10947       1.00000  0.000000E+00       1.00000  0.000000E+00      0.902533       56.0249       1.00000       11.3620  0.000000E+00     -0.624826  0.000000E+00     -0.624826  0.000000E+00  0.000000E+00       1.24965       61.3971     -0.203006        1       Scatag_D
      47       1698.45       5568.46  0.000000E+00  0.000000E+00       2288.18  0.000000E+00       2685.77       6846.43  0.000000E+00       8440.42       53.8171       7324.03 -1.000000E+10 -1.000000E+10 -1.000000E+10 -1.000000E+10 -1.000000E+10  0.000000E+00  0.000000E+00  0.000000E+00  0.000000E+00  0.000000E+00  0.000000E+00  0.000000E+00  0.000000E+00  0.000000E+00  0.000000E+00  0.000000E+00  0.000000E+00       Funtag_D
      48  5.179985E+05  4.831426E+05       34855.9       10.9385     0.0641467      0.720888      0.223690      0.232866     0.0106379 -7.753749E-05    0.00779177       1.01101       Opttag_D
      48       64.3989       4.11129       1.00000  0.000000E+00       1.00000  0.000000E+00      0.902500       56.0232       1.00000       11.3645  0.000000E+00     -0.623776  0.000000E+00     -0.623776  0.000000E+00  0.000000E+00       1.24755       61.3971     -0.203006        1       Scatag_D
      48       1697.27       5562.84  0.000000E+00  0.000000E+00       2290.53  0.000000E+00       2680.11       6829.65  0.000000E+00       8431.78       53.8259       7309.91 -1.000000E+10 -1.000000E+10 -1.000000E+10 -1.000000E+10 -1.000000E+10  0.000000E+00  0.000000E+00  0.000000E+00  0.000000E+00  0.000000E+00  0.000000E+00  0.000000E+00  0.000000E+00  0.000000E+00  0.000000E+00  0.000000E+00  0.000000E+00       Funtag_D
      49  5.179788E+05  4.831676E+05       34811.2       9.83441     0.0649300      0.738491      0.223704      0.232873     0.0105826 -1.380816E-04    0.00777910       1.01060       Opttag_D
      49       64.4023       4.11296       1.00000  0.000000E+00       1.00000  0.000000E+00      0.902485       56.0250       1.00000       11.3648  0.000000E+00     -0.623016  0.000000E+00     -0.623016  0.000000E+00  0.000000E+00       1.24603       61.3971     -0.203006        1       Scatag_D
      49       1691.76       5558.29  0.000000E+00  0.000000E+00       2292.12  0.000000E+00       2677.19       6812.66  0.000000E+00       8426.67       53.7271       7298.83 -1.000000E+10 -1.000000E+10 -1.000000E+10 -1.000000E+10 -1.000000E+10  0.000000E+00  0.000000E+00  0.000000E+00  0.000000E+00  0.000000E+00  0.000000E+00  0.000000E+00  0.000000E+00  0.000000E+00  0.000000E+00  0.000000E+00  0.000000E+00       Funtag_D
      50  5.179592E+05  4.831955E+05       34763.7       8.99605     0.0657394      0.755590      0.223720      0.232881     0.0105208 -1.891471E-04    0.00776390       1.01019       Opttag_D
      50       64.4058       4.11490       1.00000  0.000000E+00       1.00000  0.000000E+00      0.902477       56.0286       1.00000       11.3640  0.000000E+00     -0.622204  0.000000E+00     -0.622204  0.000000E+00  0.000000E+00       1.24441       61.3971     -0.203006        1       Scatag_D
      50       1685.15       5553.84  0.000000E+00  0.000000E+00       2293.77  0.000000E+00       2675.07       6794.07  0.000000E+00       8422.06       53.5608       7286.19 -1.000000E+10 -1.000000E+10 -1.000000E+10 -1.000000E+10 -1.000000E+10  0.000000E+00  0.000000E+00  0.000000E+00  0.000000E+00  0.000000E+00  0.000000E+00  0.000000E+00  0.000000E+00  0.000000E+00  0.000000E+00  0.000000E+00  0.000000E+00       Funtag_D
      51  5.179403E+05  4.832205E+05       34719.8       9.74690     0.0665890      0.772303      0.223732      0.232891     0.0104653 -2.320147E-04    0.00775265       1.00979       Opttag_D
      51       64.4088       4.11662       1.00000  0.000000E+00       1.00000  0.000000E+00      0.902464       56.0316       1.00000       11.3627  0.000000E+00     -0.621319  0.000000E+00     -0.621319  0.000000E+00  0.000000E+00       1.24264       61.3971     -0.203006        1       Scatag_D
      51       1680.27       5549.38  0.000000E+00  0.000000E+00       2295.48  0.000000E+00       2673.70       6778.68  0.000000E+00       8417.66       53.3604       7271.28 -1.000000E+10 -1.000000E+10 -1.000000E+10 -1.000000E+10 -1.000000E+10  0.000000E+00  0.000000E+00  0.000000E+00  0.000000E+00  0.000000E+00  0.000000E+00  0.000000E+00  0.000000E+00  0.000000E+00  0.000000E+00  0.000000E+00  0.000000E+00       Funtag_D
      52  5.179148E+05  4.832666E+05       34648.1       14.4754     0.0685761      0.808625      0.223755      0.232909     0.0103631 -3.336332E-04    0.00774588       1.00916       Opttag_D
      52       64.4145       4.11973       1.00000  0.000000E+00       1.00000  0.000000E+00      0.902426       56.0347       1.00000       11.3610  0.000000E+00     -0.619213  0.000000E+00     -0.619213  0.000000E+00  0.000000E+00       1.23843       61.3971     -0.203006        1       Scatag_D
      52       1677.34       5542.43  0.000000E+00  0.000000E+00       2299.44  0.000000E+00       2671.96       6760.26  0.000000E+00       8409.08       53.0291       7234.60 -1.000000E+10 -1.000000E+10 -1.000000E+10 -1.000000E+10 -1.000000E+10  0.000000E+00  0.000000E+00  0.000000E+00  0.000000E+00  0.000000E+00  0.000000E+00  0.000000E+00  0.000000E+00  0.000000E+00  0.000000E+00  0.000000E+00  0.000000E+00       Funtag_D
      53  5.178801E+05  4.832977E+05       34582.5       13.2447     0.0702897      0.837585      0.223767      0.232937     0.0102943 -4.376428E-04    0.00774902       1.00862       Opttag_D
      53       64.4183       4.12169       1.00000  0.000000E+00       1.00000  0.000000E+00      0.902387       56.0331       1.00000       11.3607  0.000000E+00     -0.617358  0.000000E+00     -0.617358  0.000000E+00  0.000000E+00       1.23472       61.3971     -0.203006        1       Scatag_D
      53       1678.70       5536.52  0.000000E+00  0.000000E+00       2302.80  0.000000E+00       2671.14       6742.71  0.000000E+00       8402.73       52.8940       7194.96 -1.000000E+10 -1.000000E+10 -1.000000E+10 -1.000000E+10 -1.000000E+10  0.000000E+00  0.000000E+00  0.000000E+00  0.000000E+00  0.000000E+00  0.000000E+00  0.000000E+00  0.000000E+00  0.000000E+00  0.000000E+00  0.000000E+00  0.000000E+00       Funtag_D
      54  5.178250E+05  4.833645E+05       34460.5       17.9622     0.0740969      0.897135      0.223805      0.233011     0.0101463 -6.837475E-04    0.00778210       1.00806       Opttag_D
      54       64.4260       4.12567       1.00000  0.000000E+00       1.00000  0.000000E+00      0.902304       56.0267       1.00000       11.3629  0.000000E+00     -0.613351  0.000000E+00     -0.613351  0.000000E+00  0.000000E+00       1.22670       61.3971     -0.203006        1       Scatag_D
      54       1693.06       5530.42  0.000000E+00  0.000000E+00       2310.26  0.000000E+00       2671.12       6733.22  0.000000E+00       8392.25       52.8177       7077.36 -1.000000E+10 -1.000000E+10 -1.000000E+10 -1.000000E+10 -1.000000E+10  0.000000E+00  0.000000E+00  0.000000E+00  0.000000E+00  0.000000E+00  0.000000E+00  0.000000E+00  0.000000E+00  0.000000E+00  0.000000E+00  0.000000E+00  0.000000E+00       Funtag_D
      55  5.177947E+05  4.833996E+05       34395.1       20.2129     0.0761582      0.926930      0.223830      0.233068     0.0100685 -8.267819E-04    0.00780580       1.00803       Opttag_D
      55       64.4301       4.12775       1.00000  0.000000E+00       1.00000  0.000000E+00      0.902264       56.0226       1.00000       11.3645  0.000000E+00     -0.611211  0.000000E+00     -0.611211  0.000000E+00  0.000000E+00       1.22242       61.3971     -0.203006        2       Scatag_D
      55       1703.39       5530.09  0.000000E+00  0.000000E+00       2314.31  0.000000E+00       2671.63       6733.89  0.000000E+00       8387.44       52.8302       7001.48 -1.000000E+10 -1.000000E+10 -1.000000E+10 -1.000000E+10 -1.000000E+10  0.000000E+00  0.000000E+00  0.000000E+00  0.000000E+00  0.000000E+00  0.000000E+00  0.000000E+00  0.000000E+00  0.000000E+00  0.000000E+00  0.000000E+00  0.000000E+00       Funtag_D
      56  5.177485E+05  4.833979E+05       34350.7       28.2667     0.0788338      0.960629      0.223829      0.233142     0.0100723 -9.873221E-04    0.00783907       1.00834       Opttag_D
      56       64.4332       4.12796       1.00000  0.000000E+00       1.00000  0.000000E+00      0.902180       56.0088       1.00000       11.3671  0.000000E+00     -0.608839  0.000000E+00     -0.608839  0.000000E+00  0.000000E+00       1.21768       61.3971     -0.203006        1       Scatag_D
      56       1717.94       5533.46  0.000000E+00  0.000000E+00       2319.34  0.000000E+00       2669.82       6782.84  0.000000E+00       8376.00       53.2005       6898.06 -1.000000E+10 -1.000000E+10 -1.000000E+10 -1.000000E+10 -1.000000E+10  0.000000E+00  0.000000E+00  0.000000E+00  0.000000E+00  0.000000E+00  0.000000E+00  0.000000E+00  0.000000E+00  0.000000E+00  0.000000E+00  0.000000E+00  0.000000E+00       Funtag_D
      57  5.176823E+05  4.833264E+05       34355.9       22.2215     0.0769132      0.932033      0.223781      0.233082     0.0102307 -8.502652E-04    0.00784904       1.00815       Opttag_D
      57       64.4286       4.12424       1.00000  0.000000E+00       1.00000  0.000000E+00      0.902195       56.0077       1.00000       11.3637  0.000000E+00     -0.611029  0.000000E+00     -0.611029  0.000000E+00  0.000000E+00       1.22206       61.3971     -0.203006        1       Scatag_D
      57       1722.31       5531.34  0.000000E+00  0.000000E+00       2315.23  0.000000E+00       2666.01       6777.14  0.000000E+00       8374.52       53.3580       6915.98 -1.000000E+10 -1.000000E+10 -1.000000E+10 -1.000000E+10 -1.000000E+10  0.000000E+00  0.000000E+00  0.000000E+00  0.000000E+00  0.000000E+00  0.000000E+00  0.000000E+00  0.000000E+00  0.000000E+00  0.000000E+00  0.000000E+00  0.000000E+00       Funtag_D
      58  5.176336E+05  4.833446E+05       34288.9       17.8978     0.0773761      0.939027      0.223795      0.233094     0.0101904 -8.794699E-04    0.00781609       1.00741       Opttag_D
      58       64.4301       4.12537       1.00000  0.000000E+00       1.00000  0.000000E+00      0.902195       56.0108       1.00000       11.3631  0.000000E+00     -0.610649  0.000000E+00     -0.610649  0.000000E+00  0.000000E+00       1.22130       61.3971     -0.203006        1       Scatag_D
      58       1707.88       5523.26  0.000000E+00  0.000000E+00       2316.22  0.000000E+00       2664.90       6758.92  0.000000E+00       8372.94       53.1602       6891.66 -1.000000E+10 -1.000000E+10 -1.000000E+10 -1.000000E+10 -1.000000E+10  0.000000E+00  0.000000E+00  0.000000E+00  0.000000E+00  0.000000E+00  0.000000E+00  0.000000E+00  0.000000E+00  0.000000E+00  0.000000E+00  0.000000E+00  0.000000E+00       Funtag_D
      59  5.175450E+05  4.833672E+05       34177.8       17.5165     0.0783589      0.951206      0.223809      0.233118     0.0101402 -9.315600E-04    0.00780019       1.00644       Opttag_D
      59       64.4326       4.12701       1.00000  0.000000E+00       1.00000  0.000000E+00      0.902188       56.0154       1.00000       11.3609  0.000000E+00     -0.610042  0.000000E+00     -0.610042  0.000000E+00  0.000000E+00       1.22008       61.3971     -0.203006        1       Scatag_D
      59       1700.94       5512.66  0.000000E+00  0.000000E+00       2318.15  0.000000E+00       2660.85       6746.80  0.000000E+00       8367.01       52.8799       6818.52 -1.000000E+10 -1.000000E+10 -1.000000E+10 -1.000000E+10 -1.000000E+10  0.000000E+00  0.000000E+00  0.000000E+00  0.000000E+00  0.000000E+00  0.000000E+00  0.000000E+00  0.000000E+00  0.000000E+00  0.000000E+00  0.000000E+00  0.000000E+00       Funtag_D
      60  5.174582E+05  4.833775E+05       34080.7       22.0606     0.0792323      0.982513      0.223815      0.233137     0.0101176 -9.817715E-04    0.00782023       1.00616       Opttag_D
      60       64.4344       4.12806       1.00000  0.000000E+00       1.00000  0.000000E+00      0.902176       56.0200       1.00000       11.3562  0.000000E+00     -0.609672  0.000000E+00     -0.609672  0.000000E+00  0.000000E+00       1.21934       61.3971     -0.203006        1       Scatag_D
      60       1709.69       5509.62  0.000000E+00  0.000000E+00       2319.62  0.000000E+00       2656.63       6752.14  0.000000E+00       8359.51       52.7181       6720.80 -1.000000E+10 -1.000000E+10 -1.000000E+10 -1.000000E+10 -1.000000E+10  0.000000E+00  0.000000E+00  0.000000E+00  0.000000E+00  0.000000E+00  0.000000E+00  0.000000E+00  0.000000E+00  0.000000E+00  0.000000E+00  0.000000E+00  0.000000E+00       Funtag_D
      61  5.174145E+05  4.833630E+05       34051.5       23.2175     0.0781531      0.989311      0.223804      0.233105     0.0101496 -8.999644E-04    0.00779993       1.00638       Opttag_D
      61       64.4320       4.12723       1.00000  0.000000E+00       1.00000  0.000000E+00      0.902192       56.0244       1.00000       11.3524  0.000000E+00     -0.610738  0.000000E+00     -0.610738  0.000000E+00  0.000000E+00       1.22148       61.3971     -0.203006        2       Scatag_D
      61       1700.82       5512.03  0.000000E+00  0.000000E+00       2317.40  0.000000E+00       2656.80       6755.55  0.000000E+00       8361.24       52.6479       6695.04 -1.000000E+10 -1.000000E+10 -1.000000E+10 -1.000000E+10 -1.000000E+10  0.000000E+00  0.000000E+00  0.000000E+00  0.000000E+00  0.000000E+00  0.000000E+00  0.000000E+00  0.000000E+00  0.000000E+00  0.000000E+00  0.000000E+00  0.000000E+00       Funtag_D
      62  5.173575E+05  4.833506E+05       34006.9       15.6548     0.0774030      0.997552      0.223795      0.233087     0.0101772 -8.667462E-04    0.00777820       1.00634       Opttag_D
      62       64.4297       4.12633       1.00000  0.000000E+00       1.00000  0.000000E+00      0.902199       56.0249       1.00000       11.3499  0.000000E+00     -0.611227  0.000000E+00     -0.611227  0.000000E+00  0.000000E+00       1.22245       61.3971     -0.203006        1       Scatag_D
      62       1691.36       5511.52  0.000000E+00  0.000000E+00       2315.80  0.000000E+00       2657.53       6733.87  0.000000E+00       8363.29       52.6527       6680.92 -1.000000E+10 -1.000000E+10 -1.000000E+10 -1.000000E+10 -1.000000E+10  0.000000E+00  0.000000E+00  0.000000E+00  0.000000E+00  0.000000E+00  0.000000E+00  0.000000E+00  0.000000E+00  0.000000E+00  0.000000E+00  0.000000E+00  0.000000E+00       Funtag_D
      63  5.173162E+05  4.833331E+05       33983.1       12.2028     0.0767422       1.00897      0.223780      0.233072     0.0102158 -8.313078E-04    0.00776392       1.00618       Opttag_D
      63       64.4268       4.12504       1.00000  0.000000E+00       1.00000  0.000000E+00      0.902200       56.0229       1.00000       11.3480  0.000000E+00     -0.611541  0.000000E+00     -0.611541  0.000000E+00  0.000000E+00       1.22308       61.3971     -0.203006        1       Scatag_D
      63       1685.16       5509.83  0.000000E+00  0.000000E+00       2314.28  0.000000E+00       2658.55       6727.42  0.000000E+00       8366.03       52.7363       6669.06 -1.000000E+10 -1.000000E+10 -1.000000E+10 -1.000000E+10 -1.000000E+10  0.000000E+00  0.000000E+00  0.000000E+00  0.000000E+00  0.000000E+00  0.000000E+00  0.000000E+00  0.000000E+00  0.000000E+00  0.000000E+00  0.000000E+00  0.000000E+00       Funtag_D
      64  5.172879E+05  4.833151E+05       33972.8       11.7357     0.0764577       1.02778      0.223768      0.233069     0.0102558 -8.192673E-04    0.00776289       1.00613       Opttag_D
      64       64.4242       4.12371       1.00000  0.000000E+00       1.00000  0.000000E+00      0.902191       56.0190       1.00000       11.3464  0.000000E+00     -0.611492  0.000000E+00     -0.611492  0.000000E+00  0.000000E+00       1.22298       61.3971     -0.203006        1       Scatag_D
      64       1684.71       5509.21  0.000000E+00  0.000000E+00       2313.38  0.000000E+00       2659.67       6728.98  0.000000E+00       8368.31       52.8692       6655.72 -1.000000E+10 -1.000000E+10 -1.000000E+10 -1.000000E+10 -1.000000E+10  0.000000E+00  0.000000E+00  0.000000E+00  0.000000E+00  0.000000E+00  0.000000E+00  0.000000E+00  0.000000E+00  0.000000E+00  0.000000E+00  0.000000E+00  0.000000E+00       Funtag_D
      65  5.172683E+05  4.833005E+05       33967.8       11.4601     0.0764989       1.04624      0.223755      0.233074     0.0102881 -8.160223E-04    0.00776475       1.00620       Opttag_D
      65       64.4226       4.12281       1.00000  0.000000E+00       1.00000  0.000000E+00      0.902182       56.0162       1.00000       11.3443  0.000000E+00     -0.611347  0.000000E+00     -0.611347  0.000000E+00  0.000000E+00       1.22269       61.3971     -0.203006        1       Scatag_D
      65       1685.52       5510.04  0.000000E+00  0.000000E+00       2313.07  0.000000E+00       2660.73       6730.73  0.000000E+00       8369.79       52.9639       6644.95 -1.000000E+10 -1.000000E+10 -1.000000E+10 -1.000000E+10 -1.000000E+10  0.000000E+00  0.000000E+00  0.000000E+00  0.000000E+00  0.000000E+00  0.000000E+00  0.000000E+00  0.000000E+00  0.000000E+00  0.000000E+00  0.000000E+00  0.000000E+00       Funtag_D
      66  5.172546E+05  4.832972E+05       33957.3       9.19635     0.0766634       1.05009      0.223751      0.233079     0.0102954 -8.251594E-04    0.00776355       1.00610       Opttag_D
      66       64.4226       4.12268       1.00000  0.000000E+00       1.00000  0.000000E+00      0.902175       56.0163       1.00000       11.3436  0.000000E+00     -0.611257  0.000000E+00     -0.611257  0.000000E+00  0.000000E+00       1.22251       61.3971     -0.203006        1       Scatag_D
      66       1685.00       5508.86  0.000000E+00  0.000000E+00       2313.24  0.000000E+00       2661.01       6727.20  0.000000E+00       8369.48       52.9443       6639.61 -1.000000E+10 -1.000000E+10 -1.000000E+10 -1.000000E+10 -1.000000E+10  0.000000E+00  0.000000E+00  0.000000E+00  0.000000E+00  0.000000E+00  0.000000E+00  0.000000E+00  0.000000E+00  0.000000E+00  0.000000E+00  0.000000E+00  0.000000E+00       Funtag_D
      67  5.172377E+05  4.832922E+05       33945.4       8.90366     0.0768962       1.05516      0.223745      0.233088     0.0103065 -8.412134E-04    0.00776178       1.00597       Opttag_D
      67       64.4226       4.12256       1.00000  0.000000E+00       1.00000  0.000000E+00      0.902166       56.0174       1.00000       11.3416  0.000000E+00     -0.611197  0.000000E+00     -0.611197  0.000000E+00  0.000000E+00       1.22239       61.3971     -0.203006        1       Scatag_D
      67       1684.23       5507.45  0.000000E+00  0.000000E+00       2313.44  0.000000E+00       2662.26       6725.22  0.000000E+00       8369.18       52.9017       6630.76 -1.000000E+10 -1.000000E+10 -1.000000E+10 -1.000000E+10 -1.000000E+10  0.000000E+00  0.000000E+00  0.000000E+00  0.000000E+00  0.000000E+00  0.000000E+00  0.000000E+00  0.000000E+00  0.000000E+00  0.000000E+00  0.000000E+00  0.000000E+00       Funtag_D
      68  5.172199E+05  4.832840E+05       33935.9       9.06120     0.0770340       1.06082      0.223737      0.233097     0.0103247 -8.686248E-04    0.00776263       1.00606       Opttag_D
      68       64.4220       4.12226       1.00000  0.000000E+00       1.00000  0.000000E+00      0.902155       56.0193       1.00000       11.3382  0.000000E+00     -0.611291  0.000000E+00     -0.611291  0.000000E+00  0.000000E+00       1.22258       61.3971     -0.203006        1       Scatag_D
      68       1684.60       5508.46  0.000000E+00  0.000000E+00       2313.37  0.000000E+00       2665.19       6722.47  0.000000E+00       8369.98       52.9221       6618.95 -1.000000E+10 -1.000000E+10 -1.000000E+10 -1.000000E+10 -1.000000E+10  0.000000E+00  0.000000E+00  0.000000E+00  0.000000E+00  0.000000E+00  0.000000E+00  0.000000E+00  0.000000E+00  0.000000E+00  0.000000E+00  0.000000E+00  0.000000E+00       Funtag_D
      69  5.171959E+05  4.832641E+05       33931.7       13.7923     0.0772083       1.06983      0.223715      0.233111     0.0103686 -9.183240E-04    0.00777183       1.00679       Opttag_D
      69       64.4202       4.12138       1.00000  0.000000E+00       1.00000  0.000000E+00      0.902124       56.0233       1.00000       11.3305  0.000000E+00     -0.611624  0.000000E+00     -0.611624  0.000000E+00  0.000000E+00       1.22325       61.3971     -0.203006        1       Scatag_D
      69       1688.59       5516.52  0.000000E+00  0.000000E+00       2313.09  0.000000E+00       2674.46       6724.85  0.000000E+00       8373.38       53.0810       6587.76 -1.000000E+10 -1.000000E+10 -1.000000E+10 -1.000000E+10 -1.000000E+10  0.000000E+00  0.000000E+00  0.000000E+00  0.000000E+00  0.000000E+00  0.000000E+00  0.000000E+00  0.000000E+00  0.000000E+00  0.000000E+00  0.000000E+00  0.000000E+00       Funtag_D
      70  5.171663E+05  4.832532E+05       33913.1       9.31151     0.0771775       1.07182      0.223704      0.233120     0.0103928 -9.651197E-04    0.00777348       1.00708       Opttag_D
      70       64.4189       4.12082       1.00000  0.000000E+00       1.00000  0.000000E+00      0.902106       56.0249       1.00000       11.3272  0.000000E+00     -0.611946  0.000000E+00     -0.611946  0.000000E+00  0.000000E+00       1.22389       61.3971     -0.203006        1       Scatag_D
      70       1689.31       5519.63  0.000000E+00  0.000000E+00       2312.83  0.000000E+00       2679.06       6716.11  0.000000E+00       8375.38       53.1997       6567.58 -1.000000E+10 -1.000000E+10 -1.000000E+10 -1.000000E+10 -1.000000E+10  0.000000E+00  0.000000E+00  0.000000E+00  0.000000E+00  0.000000E+00  0.000000E+00  0.000000E+00  0.000000E+00  0.000000E+00  0.000000E+00  0.000000E+00  0.000000E+00       Funtag_D
      71  5.171344E+05  4.832391E+05       33895.3       8.69686     0.0769815       1.07008      0.223692      0.233125     0.0104240   -0.00100397    0.00777253       1.00724       Opttag_D
      71       64.4170       4.11994       1.00000  0.000000E+00       1.00000  0.000000E+00      0.902082       56.0253       1.00000       11.3250  0.000000E+00     -0.612449  0.000000E+00     -0.612449  0.000000E+00  0.000000E+00       1.22490       61.3971     -0.203006        1       Scatag_D
      71       1688.90       5521.42  0.000000E+00  0.000000E+00       2312.45  0.000000E+00       2682.30       6718.30  0.000000E+00       8377.13       53.3665       6541.44 -1.000000E+10 -1.000000E+10 -1.000000E+10 -1.000000E+10 -1.000000E+10  0.000000E+00  0.000000E+00  0.000000E+00  0.000000E+00  0.000000E+00  0.000000E+00  0.000000E+00  0.000000E+00  0.000000E+00  0.000000E+00  0.000000E+00  0.000000E+00       Funtag_D
      72  5.171152E+05  4.832236E+05       33891.5       8.54510     0.0768640       1.07048      0.223680      0.233129     0.0104584   -0.00102968    0.00777574       1.00749       Opttag_D
      72       64.4151       4.11898       1.00000  0.000000E+00       1.00000  0.000000E+00      0.902056       56.0255       1.00000       11.3233  0.000000E+00     -0.612847  0.000000E+00     -0.612847  0.000000E+00  0.000000E+00       1.22569       61.3971     -0.203006        1       Scatag_D
      72       1690.29       5524.12  0.000000E+00  0.000000E+00       2312.19  0.000000E+00       2684.17       6723.27  0.000000E+00       8378.74       53.5038       6525.25 -1.000000E+10 -1.000000E+10 -1.000000E+10 -1.000000E+10 -1.000000E+10  0.000000E+00  0.000000E+00  0.000000E+00  0.000000E+00  0.000000E+00  0.000000E+00  0.000000E+00  0.000000E+00  0.000000E+00  0.000000E+00  0.000000E+00  0.000000E+00       Funtag_D
      73  5.171025E+05  4.832086E+05       33893.9       9.06602     0.0767573       1.07041      0.223669      0.233136     0.0104918   -0.00103087    0.00778038       1.00760       Opttag_D
      73       64.4135       4.11801       1.00000  0.000000E+00       1.00000  0.000000E+00      0.902030       56.0248       1.00000       11.3231  0.000000E+00     -0.613173  0.000000E+00     -0.613173  0.000000E+00  0.000000E+00       1.22635       61.3971     -0.203006        1       Scatag_D
      73       1692.31       5525.33  0.000000E+00  0.000000E+00       2312.08  0.000000E+00       2682.33       6731.82  0.000000E+00       8379.45       53.5743       6517.05 -1.000000E+10 -1.000000E+10 -1.000000E+10 -1.000000E+10 -1.000000E+10  0.000000E+00  0.000000E+00  0.000000E+00  0.000000E+00  0.000000E+00  0.000000E+00  0.000000E+00  0.000000E+00  0.000000E+00  0.000000E+00  0.000000E+00  0.000000E+00       Funtag_D
      74  5.170917E+05  4.832092E+05       33882.6       5.09702     0.0768000       1.07088      0.223670      0.233134     0.0104905   -0.00101431    0.00777646       1.00742       Opttag_D
      74       64.4136       4.11807       1.00000  0.000000E+00       1.00000  0.000000E+00      0.902029       56.0253       1.00000       11.3234  0.000000E+00     -0.613079  0.000000E+00     -0.613079  0.000000E+00  0.000000E+00       1.22616       61.3971     -0.203006        1       Scatag_D
      74       1690.61       5523.41  0.000000E+00  0.000000E+00       2312.15  0.000000E+00       2680.61       6728.55  0.000000E+00       8378.98       53.5325       6514.72 -1.000000E+10 -1.000000E+10 -1.000000E+10 -1.000000E+10 -1.000000E+10  0.000000E+00  0.000000E+00  0.000000E+00  0.000000E+00  0.000000E+00  0.000000E+00  0.000000E+00  0.000000E+00  0.000000E+00  0.000000E+00  0.000000E+00  0.000000E+00       Funtag_D
      75  5.170834E+05  4.832081E+05       33875.4       4.86750     0.0768580       1.07144      0.223670      0.233134     0.0104929 -9.979963E-04    0.00777369       1.00732       Opttag_D
      75       64.4135       4.11803       1.00000  0.000000E+00       1.00000  0.000000E+00      0.902021       56.0265       1.00000       11.3232  0.000000E+00     -0.612970  0.000000E+00     -0.612970  0.000000E+00  0.000000E+00       1.22594       61.3971     -0.203006        1       Scatag_D
      75       1689.40       5522.32  0.000000E+00  0.000000E+00       2312.24  0.000000E+00       2678.56       6729.55  0.000000E+00       8378.83       53.5026       6510.95 -1.000000E+10 -1.000000E+10 -1.000000E+10 -1.000000E+10 -1.000000E+10  0.000000E+00  0.000000E+00  0.000000E+00  0.000000E+00  0.000000E+00  0.000000E+00  0.000000E+00  0.000000E+00  0.000000E+00  0.000000E+00  0.000000E+00  0.000000E+00       Funtag_D
      76  5.170751E+05  4.832062E+05       33868.9       5.28303     0.0769013       1.07120      0.223669      0.233135     0.0104970 -9.811737E-04    0.00777195       1.00731       Opttag_D
      76       64.4129       4.11789       1.00000  0.000000E+00       1.00000  0.000000E+00      0.902006       56.0282       1.00000       11.3226  0.000000E+00     -0.612888  0.000000E+00     -0.612888  0.000000E+00  0.000000E+00       1.22578       61.3971     -0.203006        1       Scatag_D
      76       1688.64       5522.22  0.000000E+00  0.000000E+00       2312.37  0.000000E+00       2676.39       6730.91  0.000000E+00       8379.15       53.5083       6505.71 -1.000000E+10 -1.000000E+10 -1.000000E+10 -1.000000E+10 -1.000000E+10  0.000000E+00  0.000000E+00  0.000000E+00  0.000000E+00  0.000000E+00  0.000000E+00  0.000000E+00  0.000000E+00  0.000000E+00  0.000000E+00  0.000000E+00  0.000000E+00       Funtag_D
      77  5.170660E+05  4.832075E+05       33858.5       8.69345     0.0770190       1.07174      0.223669      0.233133     0.0104943 -9.411241E-04    0.00776892       1.00733       Opttag_D
      77       64.4117       4.11775       1.00000  0.000000E+00       1.00000  0.000000E+00      0.901975       56.0311       1.00000       11.3214  0.000000E+00     -0.612505  0.000000E+00     -0.612505  0.000000E+00  0.000000E+00       1.22501       61.3971     -0.203006        1       Scatag_D
      77       1687.33       5522.43  0.000000E+00  0.000000E+00       2312.80  0.000000E+00       2673.74       6733.04  0.000000E+00       8379.86       53.5566       6495.79 -1.000000E+10 -1.000000E+10 -1.000000E+10 -1.000000E+10 -1.000000E+10  0.000000E+00  0.000000E+00  0.000000E+00  0.000000E+00  0.000000E+00  0.000000E+00  0.000000E+00  0.000000E+00  0.000000E+00  0.000000E+00  0.000000E+00  0.000000E+00       Funtag_D
      78  5.170546E+05  4.832089E+05       33845.7       4.80131     0.0770728       1.07332      0.223670      0.233137     0.0104910 -9.366836E-04    0.00776535       1.00733       Opttag_D
      78       64.4111       4.11773       1.00000  0.000000E+00       1.00000  0.000000E+00      0.901963       56.0321       1.00000       11.3208  0.000000E+00     -0.612423  0.000000E+00     -0.612423  0.000000E+00  0.000000E+00       1.22485       61.3971     -0.203006        1       Scatag_D
      78       1685.78       5522.34  0.000000E+00  0.000000E+00       2313.01  0.000000E+00       2673.35       6727.50  0.000000E+00       8380.10       53.6037       6489.98 -1.000000E+10 -1.000000E+10 -1.000000E+10 -1.000000E+10 -1.000000E+10  0.000000E+00  0.000000E+00  0.000000E+00  0.000000E+00  0.000000E+00  0.000000E+00  0.000000E+00  0.000000E+00  0.000000E+00  0.000000E+00  0.000000E+00  0.000000E+00       Funtag_D
      79  5.170445E+05  4.832116E+05       33833.0       5.14057     0.0771392       1.07659      0.223671      0.233139     0.0104851 -9.388185E-04    0.00776074       1.00731       Opttag_D
      79       64.4106       4.11774       1.00000  0.000000E+00       1.00000  0.000000E+00      0.901954       56.0323       1.00000       11.3202  0.000000E+00     -0.612347  0.000000E+00     -0.612347  0.000000E+00  0.000000E+00       1.22469       61.3971     -0.203006        1       Scatag_D
      79       1683.78       5522.15  0.000000E+00  0.000000E+00       2313.26  0.000000E+00       2673.27       6725.00  0.000000E+00       8380.05       53.6764       6481.78 -1.000000E+10 -1.000000E+10 -1.000000E+10 -1.000000E+10 -1.000000E+10  0.000000E+00  0.000000E+00  0.000000E+00  0.000000E+00  0.000000E+00  0.000000E+00  0.000000E+00  0.000000E+00  0.000000E+00  0.000000E+00  0.000000E+00  0.000000E+00       Funtag_D
      80  5.170325E+05  4.832130E+05       33819.5       6.33468     0.0773022       1.08371      0.223671      0.233144     0.0104820 -9.434298E-04    0.00775773       1.00740       Opttag_D
      80       64.4096       4.11763       1.00000  0.000000E+00       1.00000  0.000000E+00      0.901940       56.0323       1.00000       11.3193  0.000000E+00     -0.612243  0.000000E+00     -0.612243  0.000000E+00  0.000000E+00       1.22449       61.3971     -0.203006        1       Scatag_D
      80       1682.47       5523.12  0.000000E+00  0.000000E+00       2313.72  0.000000E+00       2673.47       6723.82  0.000000E+00       8379.76       53.7859       6469.35 -1.000000E+10 -1.000000E+10 -1.000000E+10 -1.000000E+10 -1.000000E+10  0.000000E+00  0.000000E+00  0.000000E+00  0.000000E+00  0.000000E+00  0.000000E+00  0.000000E+00  0.000000E+00  0.000000E+00  0.000000E+00  0.000000E+00  0.000000E+00       Funtag_D
      81  5.170257E+05  4.832100E+05       33815.7       12.9186     0.0776781       1.09912      0.223670      0.233158     0.0104885 -9.845041E-04    0.00775814       1.00773       Opttag_D
      81       64.4081       4.11727       1.00000  0.000000E+00       1.00000  0.000000E+00      0.901925       56.0312       1.00000       11.3178  0.000000E+00     -0.612316  0.000000E+00     -0.612316  0.000000E+00  0.000000E+00       1.22463       61.3971     -0.203006        1       Scatag_D
      81       1682.65       5526.74  0.000000E+00  0.000000E+00       2314.56  0.000000E+00       2674.12       6732.41  0.000000E+00       8378.28       53.9274       6453.02 -1.000000E+10 -1.000000E+10 -1.000000E+10 -1.000000E+10 -1.000000E+10  0.000000E+00  0.000000E+00  0.000000E+00  0.000000E+00  0.000000E+00  0.000000E+00  0.000000E+00  0.000000E+00  0.000000E+00  0.000000E+00  0.000000E+00  0.000000E+00       Funtag_D
      82  5.170077E+05  4.832052E+05       33802.5       5.55236     0.0776448       1.09864      0.223667      0.233157     0.0104992 -9.851019E-04    0.00775727       1.00754       Opttag_D
      82       64.4083       4.11709       1.00000  0.000000E+00       1.00000  0.000000E+00      0.901929       56.0304       1.00000       11.3182  0.000000E+00     -0.612490  0.000000E+00     -0.612490  0.000000E+00  0.000000E+00       1.22498       61.3971     -0.203006        1       Scatag_D
      82       1682.27       5524.67  0.000000E+00  0.000000E+00       2314.42  0.000000E+00       2673.45       6725.02  0.000000E+00       8377.36       53.8697       6451.41 -1.000000E+10 -1.000000E+10 -1.000000E+10 -1.000000E+10 -1.000000E+10  0.000000E+00  0.000000E+00  0.000000E+00  0.000000E+00  0.000000E+00  0.000000E+00  0.000000E+00  0.000000E+00  0.000000E+00  0.000000E+00  0.000000E+00  0.000000E+00       Funtag_D
      83  5.169978E+05  4.831980E+05       33799.8       4.52997     0.0777053       1.10096      0.223663      0.233161     0.0105151 -9.963738E-04    0.00775965       1.00745       Opttag_D
      83       64.4083       4.11677       1.00000  0.000000E+00       1.00000  0.000000E+00      0.901930       56.0296       1.00000       11.3183  0.000000E+00     -0.612702  0.000000E+00     -0.612702  0.000000E+00  0.000000E+00       1.22540       61.3971     -0.203006        1       Scatag_D
      83       1683.30       5523.74  0.000000E+00  0.000000E+00       2314.45  0.000000E+00       2673.06       6727.40  0.000000E+00       8375.91       53.8149       6448.08 -1.000000E+10 -1.000000E+10 -1.000000E+10 -1.000000E+10 -1.000000E+10  0.000000E+00  0.000000E+00  0.000000E+00  0.000000E+00  0.000000E+00  0.000000E+00  0.000000E+00  0.000000E+00  0.000000E+00  0.000000E+00  0.000000E+00  0.000000E+00       Funtag_D
      84  5.169870E+05  4.831867E+05       33800.2       5.33493     0.0779048       1.12741      0.223658      0.233167     0.0105402   -0.00101260    0.00776552       1.00745       Opttag_D
      84       64.4077       4.11621       1.00000  0.000000E+00       1.00000  0.000000E+00      0.901922       56.0289       1.00000       11.3181  0.000000E+00     -0.613069  0.000000E+00     -0.613069  0.000000E+00  0.000000E+00       1.22614       61.3971     -0.203006        1       Scatag_D
      84       1685.85       5523.72  0.000000E+00  0.000000E+00       2314.76  0.000000E+00       2673.05       6731.99  0.000000E+00       8373.57       53.7602       6443.53 -1.000000E+10 -1.000000E+10 -1.000000E+10 -1.000000E+10 -1.000000E+10  0.000000E+00  0.000000E+00  0.000000E+00  0.000000E+00  0.000000E+00  0.000000E+00  0.000000E+00  0.000000E+00  0.000000E+00  0.000000E+00  0.000000E+00  0.000000E+00       Funtag_D
      85  5.169803E+05  4.831787E+05       33801.6       7.26520     0.0780497       1.17170      0.223653      0.233166     0.0105578   -0.00101311    0.00776913       1.00741       Opttag_D
      85       64.4071       4.11577       1.00000  0.000000E+00       1.00000  0.000000E+00      0.901910       56.0290       1.00000       11.3179  0.000000E+00     -0.613241  0.000000E+00     -0.613241  0.000000E+00  0.000000E+00       1.22648       61.3971     -0.203006        2       Scatag_D
      85       1687.42       5523.25  0.000000E+00  0.000000E+00       2314.99  0.000000E+00       2673.35       6735.59  0.000000E+00       8371.57       53.7255       6441.70 -1.000000E+10 -1.000000E+10 -1.000000E+10 -1.000000E+10 -1.000000E+10  0.000000E+00  0.000000E+00  0.000000E+00  0.000000E+00  0.000000E+00  0.000000E+00  0.000000E+00  0.000000E+00  0.000000E+00  0.000000E+00  0.000000E+00  0.000000E+00       Funtag_D
      86  5.169727E+05  4.831716E+05       33801.1       4.14782     0.0781853       1.21132      0.223650      0.233167     0.0105736   -0.00101431    0.00777263       1.00740       Opttag_D
      86       64.4066       4.11538       1.00000  0.000000E+00       1.00000  0.000000E+00      0.901899       56.0294       1.00000       11.3176  0.000000E+00     -0.613510  0.000000E+00     -0.613510  0.000000E+00  0.000000E+00       1.22702       61.3971     -0.203006        1       Scatag_D
      86       1688.94       5523.19  0.000000E+00  0.000000E+00       2315.23  0.000000E+00       2673.80       6734.71  0.000000E+00       8369.83       53.7075       6441.66 -1.000000E+10 -1.000000E+10 -1.000000E+10 -1.000000E+10 -1.000000E+10  0.000000E+00  0.000000E+00  0.000000E+00  0.000000E+00  0.000000E+00  0.000000E+00  0.000000E+00  0.000000E+00  0.000000E+00  0.000000E+00  0.000000E+00  0.000000E+00       Funtag_D
      87  5.169667E+05  4.831670E+05       33799.7       3.44374     0.0782661       1.23439      0.223646      0.233164     0.0105840   -0.00100816    0.00777382       1.00737       Opttag_D
      87       64.4061       4.11511       1.00000  0.000000E+00       1.00000  0.000000E+00      0.901886       56.0302       1.00000       11.3175  0.000000E+00     -0.613732  0.000000E+00     -0.613732  0.000000E+00  0.000000E+00       1.22746       61.3971     -0.203006        1       Scatag_D
      87       1689.46       5522.82  0.000000E+00  0.000000E+00       2315.40  0.000000E+00       2674.39       6735.01  0.000000E+00       8368.09       53.7240       6440.81 -1.000000E+10 -1.000000E+10 -1.000000E+10 -1.000000E+10 -1.000000E+10  0.000000E+00  0.000000E+00  0.000000E+00  0.000000E+00  0.000000E+00  0.000000E+00  0.000000E+00  0.000000E+00  0.000000E+00  0.000000E+00  0.000000E+00  0.000000E+00       Funtag_D

   MaxLik Scaling Cycle 0096

   Wilson plot

   Statistics in resolution bins after refinement cycle 0003
```


|  |  |
| --- | --- |
|  | - Rxpct factors plot- Rxpct factors table- <Log-Lik Gain>- Ln Sigma A- Correlation Coefficients |


```
   Iters     Tot_Funct    Xray_Funct    Geom_Funct          Grms     rmsD2init     maxD2init             R         Rfree           LLG       LLGfree     rms(bond)    rms(angle)
   Iters             K   B_IMPF_FRAG        K_MISS   B_TEMP_MISS   K_IMPF_MISS   B_IMPF_MISS        K_SOLV   B_TEMP_SOLV   K_IMPF_SOLV   B_IMPF_SOLV        B_TEMP     B_TEMP_11     B_TEMP_12     B_TEMP_22     B_TEMP_13     B_TEMP_23     B_TEMP_33      K_WILSON      B_WILSON      FGCYCLES
   Iters          BOND         ANGLE      IMPROPER       TORSION    SINTORSION      TRIGONAL        PLANAR  BCORRELATION       CONTACT     IDEALDIST        CHIRAL    SIMILARITY       OCCSSUM  UTILDISTANCE     UTILANGLE   UTILTORSION        RHOFIT        ADP-01        ADP-02        ADP-03        ADP-04        ADP-05        ADP-06        ADP-07        ADP-08        ADP-09        ADP-10      RHOFITPC            QM
      88  5.169603E+05  4.831627E+05       33797.6       4.57030     0.0783716       1.26215      0.223643      0.233160     0.0105935   -0.00100363    0.00777412       1.00734       Opttag_D
      88       64.4055       4.11484       1.00000  0.000000E+00       1.00000  0.000000E+00      0.901869       56.0314       1.00000       11.3173  0.000000E+00     -0.613974  0.000000E+00     -0.613974  0.000000E+00  0.000000E+00       1.22795       61.3971     -0.203006        1       Scatag_D
      88       1689.59       5522.51  0.000000E+00  0.000000E+00       2315.66  0.000000E+00       2675.37       6735.69  0.000000E+00       8365.39       53.7640       6439.64 -1.000000E+10 -1.000000E+10 -1.000000E+10 -1.000000E+10 -1.000000E+10  0.000000E+00  0.000000E+00  0.000000E+00  0.000000E+00  0.000000E+00  0.000000E+00  0.000000E+00  0.000000E+00  0.000000E+00  0.000000E+00  0.000000E+00  0.000000E+00       Funtag_D
   Geometry Restraint Deviations SCREEN Information

 

 The refinement has converged on gradient RMS limit (-glim).

 No -bcorrel_zeroocchydrogens_factor flag found - keeping default: will set B for OCC=0 hydrogens to    1.200 times heavy-atom B


 TLS information. Total number of groups:     2


 Group   Natoms    FixRB   FixTLS   Group Tag    Group Spec
     1     2768      Y        Y     tls1    { A|* }
     2     2758      Y        Y     tls2    { B|* }
 
NOTE BUSTER_TLS_SET tls1 { A|* }
NOTE BUSTER_TLS_T   tls1     -1.64775E-01 -3.36840E-02  2.15737E-01 -3.93502E-02 -3.35143E-02 -2.21740E-02
NOTE BUSTER_TLS_L   tls1      1.33783E+00  1.61817E+00  1.44183E+00 -3.13773E-01  8.90801E-01 -8.51731E-01
NOTE BUSTER_TLS_S   tls1      1.51047E-01 -2.84462E-01  3.13261E-01  6.85951E-02 -1.30648E-01 -2.56570E-01  7.59762E-02  1.23108E-01
NOTE BUSTER_TLS_O   tls1      2.31588E+01 -6.71772E+01  1.06797E+01
NOTE BUSTER_TLS_FIX tls1 ALL
 
NOTE BUSTER_TLS_SET tls2 { B|* }
NOTE BUSTER_TLS_T   tls2     -2.34737E-01 -9.54492E-02  3.04843E-01  1.30665E-02 -3.15132E-02  3.78123E-02
NOTE BUSTER_TLS_L   tls2      1.51690E+00  1.05873E+00  1.03399E+00  6.62845E-01  9.72762E-01  7.91924E-01
NOTE BUSTER_TLS_S   tls2      8.67167E-04  4.13351E-02 -2.05843E-01 -1.01273E-01 -1.11597E-01  4.88749E-02  1.27152E-01 -6.61129E-02
NOTE BUSTER_TLS_O   tls2     -2.22253E+01 -7.89989E+01  3.21640E+00
NOTE BUSTER_TLS_FIX tls2 ALL

 For final position, Geometry function summary
     term                           number     weight       rms        GooF      function     fn/numb
     ================================================================================================
     BOND bond lengths (angs)       5642       2.000      0.0078       0.387      1689.59       0.299
     ANGL bond angles (degs)        7656       2.000        1.01       0.601      5522.51       0.721
     TORS split-harmonic tors       1266       0.000       17.40       1.095         0.00       0.000
     SINTOR sinusoidal tors         1266       2.000       17.40                  2315.66       1.829
     PLAN general plane (angs)       958       5.000      0.0149       0.747      2675.37       2.793
     BCORrelations (angs^2)         5642      10.000       0.968       0.346      6735.69       1.194
     CONT (bad contacts)               0       5.000                                 0.00
     IDEAL(ideal-dist contact)      3897       4.000                              8365.39
     CHIRAL (gelly semiharmon)       742       5.000                                53.76
     SIM similarity restraint for NCS and/or TARGET                               6439.64
     OCCSUM restraint on sum of occupanies                                           0.00       as none defined
     DISTAN utility restraints interatomic distance                                  0.00       as none defined
     UTILANGLE utility angle restraints                                              0.00       as none defined
     UTILTOR utility restraints on torsion angles                                    0.00       as none defined
     Peptide omega torsion angles                           2.99 degs - monitor only
 
 WARNING: Have |delta/sigma| deviations > 5.0 sigma. Number of outliers for each term: 
 WARNING:         4 bond angles.  Worst is     6.2 sigs   103.58 degs A|27:CA(ILE)=A|27:C(ILE)=A|28:N.B(ASN)

 Weighted rms      0.0078         1.007  (temporary rms bond and angle for BUSTER to pick up!)
 Weighted intra-residual rms(bond) for StdProtein (excluding H)      0.0075
 Outputing geometry progress graph in plotmtv format to file:Geom_Res.mtv

 ############### Running final geometry/screen analysis (screen_final.txt)

 s/r gelly normal termination
```

Statistics and scale factors vs. cycle number
```

|  |  |  |
| --- | --- | --- |
|  | - Rxpct factors- <Log-Lik Gain>- GEOM Residuals- Ln Sigma_A_ | - Scale Factors- B Factors- Imperfection Factors |

```
 Writing F_calc(model+solvent) to complete.mtz

   Final Geometry Information


---


### 2. Final results

Statistics and scale factors vs. cycle number
```

|  |  |  |
| --- | --- | --- |
|  | - Rxpct factors- <Log-Lik Gain>- GEOM Residuals- Ln Sigma_A_ | - Scale Factors- B Factors- Imperfection Factors |

```
   Average Figure of Merit
   Structure Factor Amplitudes


 # normal termination

  Program stopped.
```

closesok message: time spent in tree = 1150.81 (user) 33.93 (sys)

---

# Normal termination

```
 Resources usage summary:
     Time spent in client ...................  1150.81 (user)       33.96 (system)
     Time spent in scripts (    27 calls) ...     2.46 (user)        0.14 (system)
     Time spent in server ...................     0.00 (user)        0.00 (system)

     Max resident memory used (kb) .......... 434904
     Number of page faults .................. 0 (major)    334294 (minor)
     Number of context switches ............. 1406 (niced)    4883 (other)
```
